# A RANKL^+^/CXCR4^+^ B cell population accumulates in bone marrow and causes age-related osteoporosis in mice

**DOI:** 10.1038/s41413-026-00525-5

**Published:** 2026-05-18

**Authors:** Jinbo Li, Jinxiao Fan, Zhenqiang Yao, Han Jiao, Rong Duan, James O. Sanders, Addisu Mesfin, Lianping Xing, Brendan F. Boyce

**Affiliations:** 1 Department of Pathology and Laboratory Medicine, University of Rochester Medical Center, Rochester, NY USA; 2 Hebei Key Laboratory of Cardiovascular Homeostasis and Aging, Hebei Medical University, Shijiazhuang, Hebei China; 3 The Key Laboratory of Neural and Vascular Biology, Ministry of Education, Hebei Medical University, Shijiazhuang, Hebei China; 4 Center for Musculoskeletal Research, University of Rochester Medical Center, Rochester, NY USA; 5 Department of Orthopaedics and Rehabilitation Medicine, University of Rochester Medical Center, Rochester, NY USA; 6 Department of Orthopaedics, University of North Carolina, Chapel Hill, NC USA; 7 Department of Orthopedics, Washington Hospital Medical Center, Washington DC, USA

**Keywords:** Osteoporosis, Diseases

## Abstract

RANKL induces bone loss in part by promoting degradation of TRAF3, levels of which decrease in murine and human bone during aging, but the major cellular sources of RANKL in bone marrow (BM) during aging are unknown. Here, we identify RANKL^+^CXCR4^+^ B cells (RCBs) as a novel major source of RANKL in murine bone. Their numbers are increased in BM of aged WT male mice and adult mice with TRAF3 conditionally deleted in mesenchymal progenitor cells (MPCs), associated with increased expression in BM of the chemokine, CXCL12, indicating that TRAF3 in MPCs limits RCB numbers in BM of young mice. During aging, TGFβ1-induced TRAF3 degradation in MPCs promotes NF-κB-mediated expression of CXCL12, associated with higher numbers of RCBs in BM where they induce bone resorption. In addition, RCBs from aged mice caused bone loss in young NSG mice and, in an ovariectomized mouse model, accelerated osteoclastic bone resorption coupled with TRAF3 reduction and RCB accumulation in BM. Consistent with these findings, administration of the FDA-approved CXCR4 antagonist, plerixafor, reduced RCB numbers in BM and increased bone mass in naturally aged and ovariectomized mice. Reduction of RCB numbers in BM could treat/prevent osteoporosis.

## Introduction

Low-level chronic inflammation occurring in the absence of overt infection during aging, termed “inflammaging”,^1^ is implicated in the pathogenesis of cancer,^2^ atherosclerosis,^3^ type II diabetes,^4^ Alzheimer’s disease,^5^ arthritis,^6^ sarcopenia^7^ and osteoporosis.^8^ Osteoporosis is characterized by decreased bone mass and strength, associated with increased bone destruction by osteoclasts and decreased bone formation by osteoblasts, leading to increased risk of fracture, morbidity and mortality in the elderly.^9^ We reported that during aging RANKL stimulates bone resorption^10^ by activating NF-κB signaling, a master regulator of inflammatory responses^11^ and bone remodeling.^12^

TNF receptor-associated factor 3 (TRAF3), an adapter protein for TNF superfamily receptors, typically inhibits NF-κB activation by promoting proteasomal degradation of NF-κB-inducing kinase.^13^ TRAF3 inhibits RANKL-induced osteoclastogenesis by suppressing NF-κB signaling^10^ and prevents TGFβ1-induced inhibition of osteoblast differentiation by limiting GSK-3β-mediated degradation of β-catenin.^14^ Notably, TRAF3 protein levels are significantly decreased in both human and murine bone during aging.^14^ Mice we generated with TRAF3 conditional knockout (cKO) in osteoclastic cells develop early onset osteoporosis because of excessive bone resorption,^10^ and mice with TRAF3 cKO in mesenchymal progenitor cells (MPCs) also develop osteoporosis due to reduced bone formation and increased resorption induced by enhanced NF-κB RelA- and RelB-mediated RANKL production by MPCs.^14^ RANKL promotes TRAF3 degradation in osteoclast progenitors, thereby causing increased bone resorption^10,14^ and implicating it in the pathogenesis of age-related osteoporosis. However, the major cellular sources of RANKL in inflammaging-associated osteoporosis remain unknown.

Inflammaging is accompanied by alterations in immune cells, in the microenvironment in lymphoid/non-lymphoid tissues and organs, and in chemokines and cytokines that mediate interactions between immune cells and the microenvironment.^15^ However, it remains largely unknown if age-related alterations of immune cells in bone marrow (BM) cause bone loss during aging. Hematopoietic stem cells (HSCs) continuously regenerate immune and blood cells and expand numerically in BM during aging, when they commit preferentially to myeloid over lymphoid cells,^16^ leading to impaired adaptive immune responses and increased susceptibility to systemic infection.^17^

B lymphocytes express RANKL,^18^ but they comprise various sub-populations, which have different functions depending upon the other factors they express. We hypothesized that there are specific B cell subsets that modulate osteoclast formation in the BM during aging. Here, we report that RANKL^+^CXCR4^+^ B cells (which we call RCBs) are the major cellular source of RANKL in BM. Their numbers are increased in BM of aged mice, associated with increased expression of CXCL12 by MPCs as a result of TGFβ1-induced degradation of TRAF3 in MPCs. We also found that RCBs are increased in the BM of ovariectomized mice. Treatment of aged and ovariectomized mice with the FDA-approved CXCR4 inhibitor, plerixafor, reduced numbers of RCBs in BM and increased bone mass, supporting our posit that these cells cause age- and ovariectomy-related bone loss.

## Results

### RCBs are the major cellular source of RANKL and accumulate in BM during aging

**Fig. 1:**
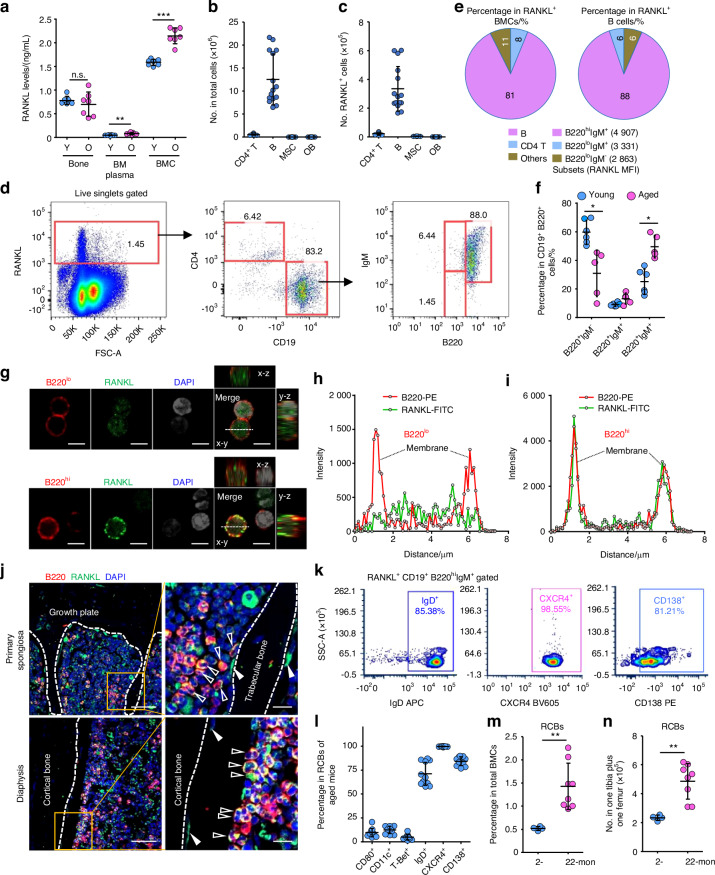
RANKL^+^CXCR4^+^B220^hi^IgM^+^ B cells (RCBs) are the major cellular source of RANKL and accumulate in BM during aging. **a** Expression levels of RANKL in protein lysates from bones (femur and tibia with BM flushed out), BM cells, and BM plasma from one leg from 7 young (2–4-month-old) and 7 old (18–22-month-old) male C57B6 mice using ELISA. **b** Numbers of CD4^+^ T cells, B cells, BM-MSCs and osteoblastic cells (OB) and **c** numbers of these cells with RANKL expression in BM from 3-month-old C57B6 mice. *n* = 15 mice. **d** Representative FACS images of RANKL^+^ BM cells enriched in B220^hi^IgM^+^ B cells. **e** Frequency of various cell types in RANKL^+^ BM cells and RANKL^+^ B cells in BM of 3-month-old C57B6 mice; *n* = 15 mice in each group. **f** % B cell subsets in total BM B cells from 2- (young) and 22-month-old (aged) C57B6 mice by FACS; *n* = 6 young and 5 aged mice. **g** Representative immunofluorescence images of decalcified frozen sections showing RANKL and B220 expression in B220^lo^ and B220^hi^ tibial BM B cells from a 22-month-old (aged) C57B6 mouse. Bar = 5 μm. Representative graphs showing changes in fluorescence intensity of B220 and RANKL from one side to the other of a B220^lo^ (**h**) and B220^hi^ (**i**) cell in (**g**). **j** Representative immunofluorescence images of decalcified frozen sections showing B220^+^ and RANKL^+^ cells, including RANKL^+^ osteoblastic cells (solid arrows) and RANKL^+^ B cells (hollow arrows), in primary spongiosa and diaphysis of tibiae of a 22-month-old (aged) C57B6 mice. Left panel: Bar = 50 μm, right panel: Bar = 10 μm. **k** Representative FACS images showing % RANKL^+^B220^hi^IgM^+^ B cells expressing IgD, CXCR4 and CD138 in BM from a 22-month-old C57B6 mouse by FACS and **l** % of these cells in 11 mice. **m** The frequency and **n** numbers of RANKL^+^CXCR4^+^B220^hi^IgM^+^CD138^+^IgD^+^ cells (RCBs) in BM of 2- and 22-month-old C57B6 mice by FACS. *n* = 4 young and 8 aged mice. Analyses: one-way ANOVA with Tukey’s post-hoc test in (**a**–**c**) and (**f**); unpaired Student’s *t* test in all others. *P < 0.05; **P < 0.01; ***P < 0.001

RANKL, expressed by various types of cells in the bone microenvironment, induces osteoclast formation as a critical step in its promotion of osteoclastic bone resorption during aging.^10^ To examine the potential cellular sources of RANKL in the bone/BM microenvironment of aged mice, we extracted protein separately from hind limb bones, BM cells and BM plasma. We found that RANKL levels were higher in BM cells than in bone or BM plasma in 2–4-month-old male C57B6 mice, and that these levels were significantly increased in BM cells from 18 to 22-month-old male mice (Fig. 1a). We did not include female mice in these studies to avoid influence of changes in female sex hormones on bone remodeling during aging as a confounding factor. To determine which cell types in bone and BM are the most abundant RANKL-expressing cells, we compared RANKL expression in CD4^+^ T cells, B cells and mesenchymal stromal cells (MSCs) in BM and in osteoblastic cells digested from bone from 3-month-old C57B6 male mice (Fig. S1a). We found significantly higher percentage of RANKL^+^ cells in MSCs than in B cells, CD4^+^ T cells or osteoblastic cells in these samples (Fig. S1b), but significantly more B cells than other cells (Fig. 1b; Fig. S1c), and that B cells were the most abundant RANKL^+^ cells in these samples (Fig. 1c). CXCL12-abundant reticular (CAR) cells, a subtype of MSCs, express RANKL and play an important role in osteoclast formation.^19^ We confirmed that BM cells expressing Nestin, a marker of CAR cells,^20^ express RANKL and, although they were located adjacent to bone surfaces in BM of aged mice, most RANKL^+^ cells were Nestin-negative cells (Fig. S1d). Notably, we next found that 80% of the RANKL^+^ BM cells were CD19^+^ B cells, of which over 80% co-expressed B220^++(hi)^ and IgM^+^ (Fig. 1d, e; Fig. S2a–c). In addition, RANKL expression by these B220^hi^IgM^+^ B cells was significantly higher than that by other B cell subsets, including pre/pro-B cells (B220^+(low)^IgM^-^) and immature B cells (B220^low^IgM^+^) (Fig. 1e; Fig. S2e, f), suggesting that they are the predominant RANKL-expressing cell population in BM of 3-month-old C57B6 male mice. Importantly, the proportion of B220^hi^IgM^+^ B cells in total BM B lymphocytes was significantly higher in 22-month-old than in 2-month-old mice, while that of pro/pre-B cells was lower (Fig. 1f). Although neutrophils are the most abundant cells in BM and stimulated neutrophils express RANKL in vitro,^21^ neutrophils are not the most abundant source of RANKL in periodontitis,^22^ and because we found that ~80% of RANKL-expressing cells in BM are B cells, we did not include neutrophils in these analyses.

Both soluble and membrane-bound RANKL stimulate osteoclast formation,^23,24^ but membrane-bound RANKL has been reported to be a more potent inducer of osteoclast formation than soluble RANKL.^24^ We found that the level of RANKL expression was significantly higher in BM B220^high^ than B220^low^ B cells in BM of aged mice (Fig. 1g–i). In addition, unlike the BM B220^low^ B cells that mainly had intracellular expression of RANKL, the B220^high^ B cells had RANKL expression predominantly on their cell membranes (Fig. 1g–i). Notably, we found that membrane-bound RANKL-expressing B cells were present in clusters and located close to bone surfaces in BM of aged mice (Fig. 1j), suggesting a positive role for these membrane-bound RANKL-expressing B220^high^ B cells in osteoclast formation during aging. However, the relative importance of these B cells, as a cellular source of RANKL, compared with other cell types, including CAR cells, is not clear from our findings and will require further study.

A subset of CD11c^+^T-bet^+^CD80^+^ B cells accumulates during parasitic, bacterial and viral infections in mouse models and in humans with autoimmune diseases,^25^ and during aging in mice and humans, as so-called age-associated B cells (ABCs).^25,26^ We found that RANKL^+^B220^hi^IgM^+^ B cells in BM from aged mice rarely expressed CD11c, T-bet, or CD80, while almost all of them expressed the chemokine receptor, CXCR4, and expressed high levels of IgD and intermediate levels of CD138 (a marker of plasma and other cells) (Fig. 1k, l; Fig. S2d). Of note, ~100% of RANKL^+^ B cells expressed CXCR4 protein (Fig. 1l), suggesting that they are different from ABCs and may potentially be attracted to BM by certain CXCR4 ligands. In addition, RANKL expression by these CXCR4^+^IgD^+^ mature (B220^hi^IgM^+^) B cells was significantly higher than that by other B cell subsets, including CXCR4^-^IgD^-^ pre/pro-B cells, total pre/pro-B cells (B220^+(low)^IgM^-^) and immature B cells (B220^low^IgM^+^) in BM (Fig. S2e, f), suggesting that RANKL^+^CXCR4^+^ B cells are the predominant RANKL-expressing cell population in BM. We have called these RANKL^+^CXCR4^+^ B220^hi^CD138^+^IgM^+^IgD^+^ B cells RCBs for short. The frequency and numbers of RCBs were significantly higher in BM from aged than from young mice (Fig. 1m, n).

### RCBs stimulate osteoclast formation and bone erosion

**Fig. 2:**
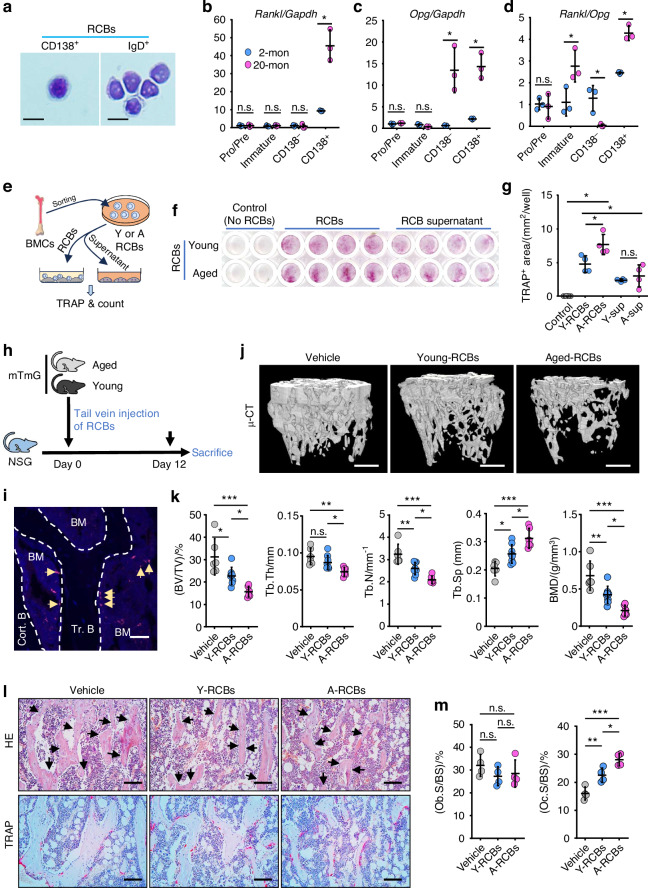
RCBs stimulate OC formation and bone loss in mice. **a** H&E-stained CD138^+^ and IgD^+^ RCBs following cytospinning. Bar = 10 μm. **b**–**d** Relative *Rankl* and *Opg* levels in bulk RNA from Pro/Pre-B (B220^+^IgM^-^), Immature B (B220^+^IgM^+^), CD138^-^ B cells (CD138^-^B220^hi^IgM^+^IgD^+^) and CD138^+^ RCBs (CD138^+^RANKL^+^B220^hi^IgM^+^IgD^+^) in BM of 2- and 20-month-old C57B6 mice. *n* = 3 samples. **e**, **f** Experimental design for RCB treatment of OCPs (generated from 2.5 × 10^4^ M-CSF-treated BM cells) from 1-month-old C57B6 mice cultured alone or with RCBs (3 × 10^4^) or RCB cell culture supernatant (sup; 100 μL) from BM of 2- (Young) and 22-month-old (Aged) male C57B6 mice for 2 d and then TRAP-stained. **g** Area of TRAP-positive cells. *n* = 4 samples in each group. **h** Experimental design for tail vein injection of RANKL^+^B220^hi^IgM^+^ B cells (RCBs; ~100% of RANKL^+^B220^hi^IgM^+^ B cells express CXCR4) from young (3-month-old) or aged (20-month-old) ROSA^mTmG^ male mice into 3-month-old male NSG recipient mice and sacrificed 12 d later. **i** Immunofluorescence image of donor-derived RCBs (tdTomato red) adjacent to bone surfaces (arrowed) in BM of tibial cryosections from an NSG recipient. Bar = 25 μm. **j** Representative images showing μCT 3D reconstruction of proximal tibial trabecular bone in NSG mice injected with no cells (Vehicle), RCBs (RANKL^+^B220^hi^IgM^+^ B cells) from young mice (Y-RCBs) or aged mice (A-RCBs), Bar = 500 μm, and **k** their mean trabecular bone volume (BV/TV), thickness (Tb.Th), number (Tb.N), separation (Tb.Sp) and bone mineral density (BMD) values. *n* = 6, 8 and 6 mice for vehicle, Y-RCB and A-RCB groups, respectively. **l** H&E and TRAP-stained paraffin sections showing osteoblasts (black arrows) and osteoclasts (red) on bone surfaces, respectively. **m** Osteoclast and osteoblast surfaces. Bar = 100 μm. *n* = 4 mice in each group. One-way ANOVA with Tukey’s post-hoc test in all experiments. *P < 0.05; **P < 0.01; ***P < 0.001

We next found that FACS-sorted CD138^+^ RCBs (RANKL^+^CXCR4^+^B220^hi^IgM^+^) and IgD^+^ RCBs were morphologically similar (Fig. 2a). Thus, we sorted CD138^+^ RCBs and compared them with CD138^-^ recirculating B cells (CD138^-^B220^hi^IgM^+^IgD^+^), immature B cells (B220^low^IgM^+^) and pre/pro-B cells (B220^low^IgM^-^) from BM of young (2-month-old) and old (20-month-old) mice. We found that mRNA levels of *TNFSF11*, the gene encoding RANKL (*Rankl)*, were highest in CD138^+^ RCB bulk RNA extracts from 2-month-old mice, and they were much higher in 20-month-old mice (Fig. 2b). In contrast, mRNA levels of *TNFRSF11B*, the gene encoding osteoprotegerin (*Opg*), were higher in both CD138^+^ RCBs and CD138^-^ recirculating B cells from old than from young mice (Fig. 2c). Of note, *Rankl*/*Opg* ratios were higher in CD138^+^ RCBs, but lower in CD138^-^ recirculating B cells from old than from young mice (Fig. 2d).

To test the osteoclast-inducing potential of RCBs, we sorted RCBs (RANKL^+^CXCR4^+^B220^hi^IgM^+^) from BM of young (2-month-old) and aged (22-month-old) mice, and separately collected RCBs and their culture supernatant for further coculture with osteoclast precursors (Fig. 2e). Importantly, in co-cultures with M-CSF-treated osteoclast precursors from young mice, RCBs sorted from young mice induced osteoclast formation without the addition of RANKL; this effect was significantly higher in co-cultures of RCBs from aged mice with osteoclast precursors from young mice, but not in cultures with conditioned medium from RCBs (Fig. 2f, g), suggesting that RCB RANKL is mainly membrane-bound.

To determine if RCBs induce bone resorption in vivo, we sorted RCBs (RANKL^+^B220^hi^IgM^+^) without CXCR4 surface Ab labeling because CXCR4 expression was observed in nearly 100% of RANKL^+^B220^hi^IgM^+^ cells and, importantly, Ab binding to CXCR4 might prevent the natural chemotactic activity of RCBs to the local bone microenvironment. Thus, we injected 5 × 10^5^ sorted RCBs (RANKL^+^B220^hi^IgM^+^) from young (3-month-old) or aged (20-month-old) ROSA^mTmG^ male mice into 3-month-old male NSG mice via tail vein and sacrificed the recipients 12 d post transfer (Fig. 2h). We first confirmed that tdTomato^+^ donor-derived B cells were present in BM of NSG recipient mice. We found that some of the tdTomato^+^ B cells were located close to the bone surfaces and this cell-to-bone surface distance was comparable between the recipient mice injected with young and aged RCBs (Fig. 2i; Fig. S3a–c), and that donor-derived RCBs maintained high expression of RANKL in the BM of NSG recipients (Fig. S3d). Of note, NSG recipients that received RCBs from either young or aged mTmG mice had lower trabecular bone mass, thickness and number, and lower bone mineral density than NSG control mice without cell transfer (Fig. 2j, k). However, the NSG recipients of RCBs from aged mice had significantly more bone loss than NSG recipients of RCBs from young mice (Fig. 2j, k). Histomorphometric analysis revealed that the bone loss in NSG recipients following RCB injection was associated with increased osteoclast, but normal osteoblast surfaces (Fig. 2l, m; Fig. S3e, f).

To further examine the importance of RANKL expression by RCBs from aged mice in triggering bone loss in NSG mice, we sorted RANKL^+^B220^hi^IgM^+^ cells (called R^+^BCs) and RANKL^-^B220^hi^IgM^+^ B cells (called R^-^BCs) from BM of aged C57B6 male mice, injected them into 3-month-old male NSG mice, and the recipients were sacrificed 12 d post transfer (Fig. S4a). We found that NSG recipients that received R^+^BCs had significantly lower trabecular bone volume, thickness and number, and lower bone mineral density than NSG mice given R^-^BCs from aged mice (Fig. S4b, c), associated with a significant decrease in numbers of F4/80^+^ macrophages in BM of recipients (Fig. S4d). In addition, body weight, liver and renal toxicity indicators, including serum alanine aminotransferase, aspartate aminotransferase, creatinine and blood urea nitrogen levels were comparable between R^+^BC and R^-^BC-injected NSG recipients (Fig. S4e, f). These findings suggest that a single transfer of RCBs was sufficient to cause significant reduction in trabecular bone mass and in macrophages in BM.

### Aged WT mice, adult mice lacking TRAF3 in MPCs and elderly humans have increased CXCL12 expression by MPCs

**Fig. 3:**
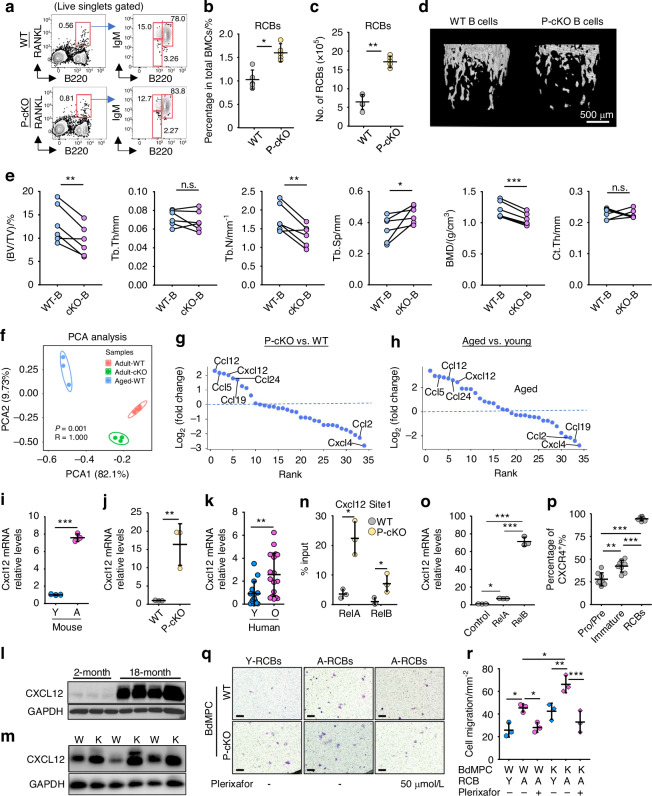
Aged WT mice and adult mice lacking TRAF3 in MPCs have increased CXCL12 expression by MPCs. **a** Representative FACS images showing the % of RANKL^+^B220^hi^IgM^+^ B cells in BM from 12-month-old TRAF3^fl/fl^ (WT) and Prx1^Cre^TRAF3^fl/fl^ (P-cKO) mice. **b**, **c** Frequency and numbers of RCBs (RANKL^+^CXCR4^+^B220^hi^IgM^+^CD138^+^IgD^+^ cells) in BM from 12-month-old WT and P-cKO mice; *n* = 5 mice. **d** Representative images showing μCT 3D reconstruction of proximal tibial trabecular bone in NSG mice injected with 8 × 10^5^ B cells magnetically sorted from BM of 12-month-old male WT or P-cKO mice, and **e** their mean values for trabecular bone volume (BV/TV), thickness (Tb.Th), number (Tb.N), separation (Tb.Sp), bone mineral density (BMD) and cortical bone thickness (Ct.Th). *n* = 6 mice for each group. **f** Principal component analysis (PCA) of mouse chemokine levels in bulk RNA extracted from CD45^-^ cells isolated from BM of 9-month-old WT and P-cKO mice and 24-month-old WT mice; *n* = 4, 3 and 3 mice, respectively. The first and second principal components explain 82.1% and 9.73%, respectively, of the total variance in the dataset. P: statistical significance; R: correlation coefficient. Chemokine screens based on their transcription levels normalized to *Gapdh* in CD45^-^ BM cells from (**g**) 9-month-old P-cKO mice (vs. 9-month-old WT mice) and (**h**) 24-month-old WT mice (vs. 9-month-old WT mice). **i**, **j** *Cxcl12* mRNA levels normalized to *Gapdh* in bulk RNA from 3rd passage BM-MSCs from 3- (Y) and 22-month-old (A) male C57B6 mice, and from 12-month-old WT and P-cKO mice. *n* = 3 samples. **k** *Cxcl1*2 mRNA levels in bulk RNA from vertebral specimens from 16 young (Y) and 18 older (O) humans, normalized to *Gapdh*. **l** WBs of BM lysates from 2- and 18-month-old male C57B6 mice and **m** from 12-month-old WT (W) and P-cKO (K) male mice. **n** Sheared chromatin from WT and P-cKO mouse bone-derived MPCs (BdMPCs) used to perform DNA IP with RelA and RelB Abs or IgG control. RT-PCR using designed primers with putative κB binding sites in the *Cxcl12* gene promotor, normalized to input. *n* = 3 samples. **o** Calvarial pre-osteoblasts from 7-d-old C57B6 mice infected with GFP control, RelA, or RelB retroviruses for 48 h, then *Cxcl12* mRNA expression tested by real-time PCR, normalized to *Gapdh*. *n* = 3 biologically independent samples. **p** CXCR4 expression on B cell subsets using FACS. **q** FACS-sorted RCBs (RANKL^+^B220^hi^IgM^+^) from 3- and 24-month-old mice were treated with vehicle or 50 μM plerixafor for 2 h, then cultured in transwell plates coated with BdMPCs from 15-month-old WT or P-cKO mice, treated with vehicle or plerixafor for 6 h, and stained with crystal violet, and **r** numbers of cells that migrated through and remained on the bottom side of transwell membranes. Bar = 100 μm. *n* = 3 independent experiments. Analyses: unpaired Student’s *t* test in (**b**, **c**) and (**i**–**k**); paired Student’s *t* test in (**e**); one-way ANOVA with Tukey’s post-hoc test in all others. *P < 0.05; **P < 0.01; ***P < 0.001

TRAF3 negatively regulates NF-κB signaling, and levels of TRAF3 fall in human and murine bone during aging.^14^ Mice with TRAF3 deleted specifically in mesenchymal lineage cells (Prx1^Cre^;TRAF3^fl/fl^; called P-cKO mice) develop early onset osteoporosis due to increased bone resorption and reduced bone formation.^14^ To determine if the P-cKO mice have increased RCBs in BM to help explain their reduced bone mass, we first confirmed that 12-month-old P-cKO mice have significantly reduced trabecular bone mass (Fig. S5a–c). We found that they have a higher percentage of recirculating B cells (B220^hi^IgM^+^) in BM (Fig. S5d) than WT littermates. Of note, like aged WT mice, 12-month-old P-cKO mice have a significantly higher percentage and number of RCBs (RANKL^+^CXCR4^+^ B220^hi^IgM^+^) than WT littermates (Fig. 3a–c), suggesting that TRAF3 in mesenchymal lineage cells limits both the accumulation of RCBs in BM and bone loss in adult mice. To further determine if these changes in B cells in BM of P-cKO mice are sufficient to cause the bone loss in these mice, we sorted B cells from BM of 12-month-old WT and P-cKO mice and injected them into 3-month-old NSG mice. We found that the NSG mice injected with B cells from P-cKO mice had significantly lower trabecular bone volume and number, lower bone mineral density and higher trabecular separation than mice injected with B cells from WT mice (Fig. 3d, e), suggesting that the increased numbers of RANKL^+^ B cells in P-cKO mice are sufficient to cause the reduction in bone volume, independent of RANKL expression by MSCs in these mice.

Various hematopoietic/immune cells are attracted to or maintained in BM by signaling through chemokine/chemokine receptors whose expression is regulated by transcription factors, particularly NF-κB.^27^ NF-κB signaling is increased in MPCs of aged WT mice and adult TRAF3 P-cKO mice.^14^ To explore potential NF-κB-induced chemotaxis signals shared by mesenchymal lineage cells from adult P-cKO and aged WT mice that might promote RCB accumulation in BM, we isolated CD45^-^ cells from BM of adult (9-month-old) WT and P-cKO mice and from aged (24-month-old) WT mice and examined the expression levels of 38 chemokines by qPCR. Principal component analysis (PCA) of chemokine mRNA levels revealed distinct clusters for 3 different groups, suggesting that they have distinct transcription profiles (Fig. 3f). The transcription levels of *Ccl12*, *Ccl24*, *Ccl5* and *Cxcl12* were significantly higher in CD45^-^ BM cells from 9-month-old P-cKO mice than in age-matched WT mice (Fig. 3g), and they were also higher in CD45^-^ BM cells from aged than from adult WT mice (Fig. 3h). Complementing the high expression of CXCR4, a receptor for CXCL12, on RCBs (Fig. 1k, l), we found that CXCR4 expression by RCBs increased with age (Fig. S5e), suggesting that RCBs might accumulate in the BM with age following chemokine attraction by CXCL12. In addition, *Cxcl12* transcription levels were significantly higher in bulk RNA from CD45^-^ BM cells than from CD45^+^ BM cells and cortical bone (Fig. S5f), suggesting that CD45^-^ cells likely play a more important role than other BM cells in the accumulation of RCBs in BM. Notably, *Cxcl12* mRNA levels were higher in MPCs from aged WT than from young WT mice (Fig. 3i), and they were also higher in MPCs from adult P-cKO mice than their WT littermates (Fig. 3j); they were also higher in human vertebral specimens from elderly subjects than from children (Fig. 3k). In addition, CXCL12 protein levels were significantly higher in BM protein lysates from 18-month-old than from 2-month-old mice (Fig. 3l), and they were also higher in BM from adult (12-month-old) P-cKO mice than from WT littermates (Fig. 3m), reflecting increasing CXCL12 protein levels in the control mice during aging. Consistent with this, there were significantly more CXCL12^+^ cells in proximal tibial metaphyses from aged WT (Fig. S5g, h) and adult P-cKO mice (Fig. S5i, j) than from their respective controls (young WT and adult WT mice, respectively).

To determine if NF-κB regulates *Cxcl12* expression in MPCs, potential κB binding sites on the *Cxcl12* gene promotor were identified and confirmed using ChIP assays. We then identified 1 κB binding site within 500 bp from the start codon of the *Cxcl12* promoter (Fig. S5k). ChIP assay data supported higher NF-κB RelA and RelB binding to this site in the *Cxcl12* gene promotor in MPCs from P-cKO mice than from WT mice (Fig. 3n). In addition, *Cxcl12* mRNA levels were significantly higher in MPCs infected with pMX-RelA or -RelB lentiviruses than in cells infected with pMX-GFP lentiviruses (Fig. 3o). Further, CXCR4 expression by RCBs was markedly higher than in Pro/Pre- (B220^+^IgM^-^) and immature (B220^+^IgM^+^) B cell subsets (Fig. 3p). To determine if the increased CXCL12 expression by MPCs from P-cKO mice results in attraction of more RCBs, we plated FACS-sorted RCBs (RANKL^+^B220^hi^IgM^+^) in a Transwell Migration System. We found that more RCBs from aged mice migrated toward WT bone-derived MPCs than RCBs from young mice, and more RCBs migrated towards bone-derived MPCs from P-cKO mice than from WT littermates. This increase in cell migration was prevented by pre-treatment of RCBs with the CXCR4 antagonist, plerixafor, and by addition of plerixafor to the culture medium (Fig. 3q, r).

### Prevention of IAP-mediated TRAF3 degradation caused by TGFβ1 inhibits RCB accumulation in BM and bone loss

**Fig. 4:**
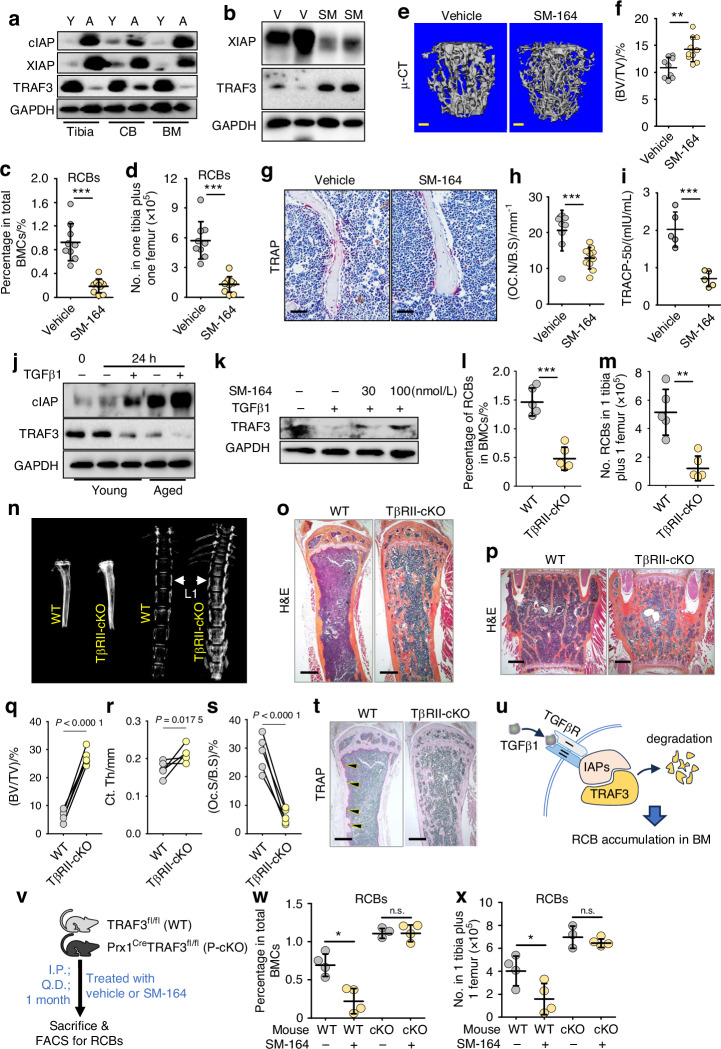
RCB accumulation in BM and bone loss caused by TGFβ1-induced TRAF3 degradation in MPCs are reduced by the IAP inhibitor, SM-164. **a** WB of cIAP, XIAP, TRAF3 and GAPDH protein expression in tibiae, femoral cortical bone, and BM cells from 3- (Y) and 22-month-old (A) C57B6 mice. **b** WB of XIAP, TRAF3 and GAPDH proteins in bones of aged mice treated with vehicle (V) or the IAP inhibitor, SM-164 (SM; 3 mg/kg/d injected intraperitoneally) once/d for 1 month. **c**, **d** Frequency and numbers of RCBs (RANKL^+^CXCR4^+^B220^hi^IgM^+^CD138^+^IgD^+^ cells) in BM of vehicle- and SM-164-treated aged mice. *n* = 9 and 10 mice, respectively. **e**, **f** Representative images showing μCT 3D reconstruction of L1 vertebral trabecular bone from vehicle- and SM-164-treated aged mice and trabecular bone volumes in the samples. Bar = 300 μm. *n* = 9 and 10 mice, respectively. **g**, **h** TRAP-stained paraffin sections of L2 vertebrae from vehicle- and SM-164-treated aged mice and OC number. Bar = 50 μm. *n* = 9 and 10 mice, respectively. **i** Serum TRACP-5b levels measured using ELISA. *n* = 5 mice. **j** WB of cIAP, TRAF3 and GAPDH proteins in MPCs from 3- and 22-month-old C57B6 mice following treatment with vehicle or 2 ng/mL TGFβ1 for 24 h. **k** WB of TRAF3 and GAPDH proteins in MPCs treated with vehicle or 2 ng/mL TGFβ1 plus increasing doses of SM-164 for 24 h. **l**, **m** Frequency and numbers of RCBs in total BM cells of 15-month-old TGFβRII^f/f^ (WT) and TGFβRII^f/f^;Prx1^Cre^ (TβRII-cKO) mice. *n* = 5 mice. **n** Radiographs of tibiae and spines (with L1 arrowed) of 15-month-old WT and TβRII-cKO mice. **o**, **p** H&E-stained sections of tibiae and L2 vertebrae of 15-month-old WT and TβRII-cKO mice. Bar = 400 μm. **q**, **r** µCT analysis of tibial trabecular bone volume (BV/TV) and cortical bone thickness (Ct.Th) values. *n* = 5 mice for each group. **s**, **t** Osteoclast surface values and representative images of TRAP-stained tibial paraffin sections of WT and TβRII-cKO mice with arrowheads pointing to endosteal osteoclasts. Bar = 400 μm. **u** Cartoon of TGFβ/IAP-mediated TRAF3 degradation. **v** Experimental design for treating 12-month-old WT and P-cKO mice with SM-164. **w**, **x** Frequency and numbers of RCBs (RANKL^+^CXCR4^+^B220^hi^IgM^+^CD138^+^IgD^+^ cells) in BM of 12-month-old WT and P-cKO mice treated with vehicle or SM-164. *n* = 4, 4, 3 and 4 mice, respectively. Analyses: one-way ANOVA with Tukey’s post-hoc test in (**w**, **x**); unpaired Student’s *t* test in all others. *P < 0.05; **P < 0.01; ***P < 0.001

We next examined if prevention of TRAF3 degradation inhibits RCB recruitment to BM during aging. We reported that the inhibitor of apoptosis proteins (IAPs), XIAP and cIAP, interact with TRAF3 protein and promote its ubiquitination and lysosomal degradation.^14^ We first confirmed that TRAF3 protein levels were markedly lower in tibiae, cortical bone, and BM of aged mice than young mice, which corresponded with increased protein levels of XIAP and cIAP (Fig. 4a). To further investigate the potential effects of inhibition of IAPs on TRAF3 and RCBs, we next treated aged mice with the bivalent second mitochondria-derived activator of caspases (Smac) mimetic IAP inhibitor, SM-164.^28,29^ We found that these mice had significantly lower XIAP and higher TRAF3 protein expression levels in bone than vehicle-treated mice (Fig. 4b), with a slight increase in body weight (Fig. S6a) and no effects on BM cellularity (Fig. S6b). SM-164-treated aged mice had a slightly expanded LSK pool, in which the frequency of long-term hematopoietic stem cells (LT-HSCs) was lower, but that of the multipotent progenitors, MPP3 and MPP4, was slightly higher than in vehicle-treated mice (Fig. S6c–e). Of note, the frequency and numbers of B220^hi^IgM^+^ B cells and RCBs were significantly lower in SM-164- than in vehicle-treated aged mice (Fig. 4c, d; Fig. S6f–h). SM-164 significantly inhibited Cxcl12 transcription in CD45^-^ BMCs from aged mice (Fig. S6i). Consistent with this, SM-164-treated aged mice had significantly increased trabecular bone volume (Fig. 4e, f; Fig. S6j, k) and increased dynamic bone formation parameters (Fig. S6l, m), associated with a significant reduction in osteoclast numbers and surfaces (Fig. 4g, h; Fig. S6n) and an increase in osteoblast numbers and surfaces (Fig. S6o, p). TRACP-5b serum levels were lower, and osteocalcin serum levels were higher in SM-164- than in vehicle-treated aged mice (Fig. 4i; Fig. S6q).

We reported that TGFβ1 induces association of IAPs and TRAF3 with the TGFβ receptor (TGFβR) and promotes TRAF3 degradation in MPCs.^14^ Consistent with this, TGFβ1 increased cIAP protein levels in MPCs from 3-month-old WT mice and to significantly higher levels in MPCs from 22-month-old WT mice, associated with a significant reduction in TRAF3 protein levels (Fig. 4j). SM-164, partially prevented the TRAF3 protein reduction in MPCs induced by TGFβ1 (Fig. 4k), suggesting that TGFβ1 signaling promotes IAP-mediated TRAF3 degradation in MPCs.

We reported that deletion of TGFβRII specifically in mesenchymal lineage cells in Prx1^Cre^TGFβRII^fl/fl^ (TGFβRII-cKO) mice effectively inhibited TRAF3 degradation.^30^ We found that 15-month-old TGFβRII-cKO mice had similar LSK pool sizes and B cell populations in BM as TGFβRII^fl/fl^ (WT) littermates (Fig. S7a–e). Of note, the frequency and numbers of RCBs in BM were significantly lower in 15-month-old TGFβRII-cKO mice than in WT littermates (Fig. 4l, m; Fig. S7f), associated with a significant increase in bone mineral density (Fig. 4n) and trabecular bone volume in tibiae (Fig. 4o) and vertebrae (Fig. 4p). μCT analysis revealed that the tibial trabecular bone volume and cortical thickness of TGFβRII-cKO mice were significantly higher than in WT littermates (Fig. 4q, r), associated with a significant decrease in osteoclast numbers in TGFβRII-cKO mice (Fig. 4s, t).

Given that the size of the marrow space is reduced in TGFβRII-cKO mice, the numbers of RCBs, corrected for the reduction in BM space due to the increased bone mass in the TGFβRII-cKO mice, were significantly lower in 15-month-old TGFβRII-cKO than WT mice (Fig. S7g, h), suggesting that TGFβRII deletion in MPCs prevented RCB accumulation during aging. TGFβ1 promotes IAP-mediated TRAF3 degradation, leading to an increase in RCB accumulation in BM during aging (Fig. 4u). To further determine if the beneficial effects of targeting IAP activity depend on restoration of TRAF3, we treated WT and Prx1^Cre^;TRAF3^fl/fl^ (P-cKO) mice with SM-164 (Fig. 4v). The frequency and numbers of RCBs were significantly reduced in WT mice given SM-164, but were not in age-matched P-cKO mice (Fig. 4w, x), suggesting that TRAF3 expression by mesenchymal lineage cells is required for the RCB reduction caused by SM-164 treatment.

### The CXCR4 antagonist, plerixafor, prevents RCB accumulation in BM and increases bone mass in aged male mice

**Fig. 5:**
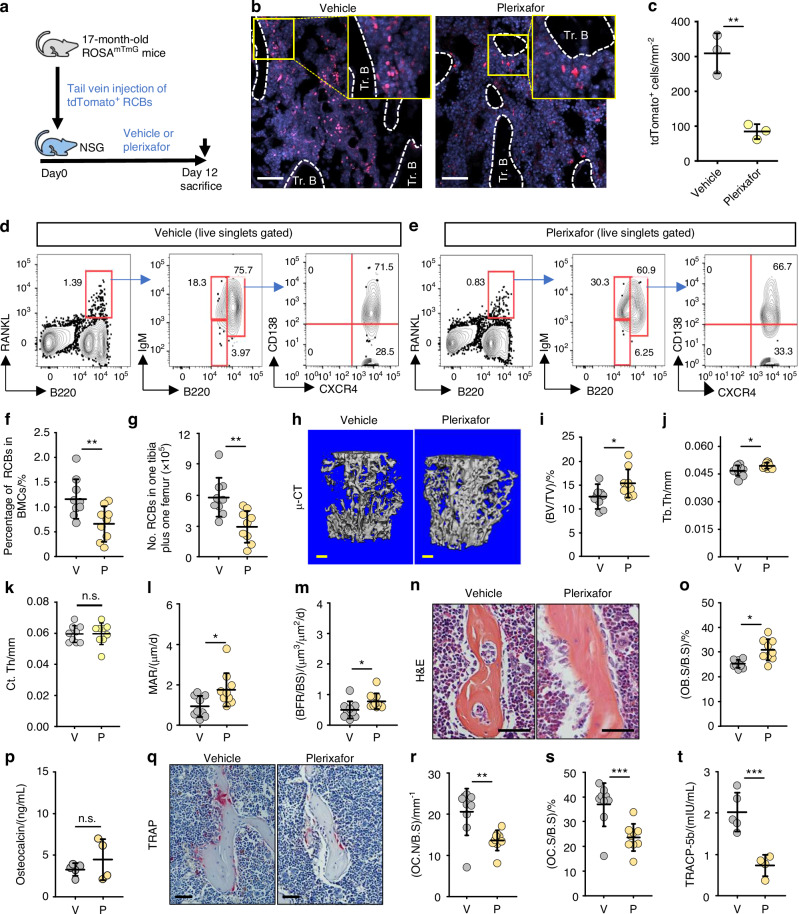
The CXCR4 antagonist, plerixafor, prevents RCB accumulation in BM and increases bone mass in aged mice. **a** Experimental design for transferring magnetically sorted RCBs (RANKL^+^B220^hi^IgM^+^) B cells from 17-month-old ROSA^mTmG^ mice into 3-month-old NSG mice and subsequently treating these NSG recipients with plerixafor. **b**, **c** Representative images showing ROSA^mTmG^ mouse-derived tdTomato^+^ cells (red) in metaphysis of tibiae from NSG mice treated with vehicle or plerixafor, and **c** the number of tdTomato^+^ cells. Bar = 50 μm. *n* = 3 mice for each group. **d**, **e** Representative FACS images showing the frequency of RCBs (RANKL^+^CXCR4^+^B220^hi^IgM^+^CD138^+^) in BM from 22-month-old male C57B6 mice treated with vehicle (V) or plerixafor (P; 5 mg/kg) once/d intraperitoneally for 1 month. **f**, **g** Frequency and numbers of RCBs (RANKL^+^CXCR4^+^B220^hi^IgM^+^CD138^+^) in BM of vehicle- (V) or plerixafor- (P) treated aged mice. *n* = 9 mice for each group. **h** Representative images of μCT 3D reconstruction of L1 vertebrae, and analysis of **i** trabecular bone volume, **j** thickness and **k** cortical bone thickness. Bar = 1 mm. *n* = 9 mice for each group. **l**, **m** Mineral apposition rate (MAR) and bone formation rate (BFR) in L1 vertebrae. *n* = 9 mice for each group. **n** H&E-stained sections of L2 vertebrae and **o** Osteoblast surfaces. Bar = 50 μm. *n* = 9 mice for each group. **p** Serum osteocalcin levels tested by ELISA. *n* = 5 and 4 mice, respectively. **q** TRAP-stained paraffin sections of L2 vertebrae and **r** osteoclast numbers and **s** surfaces on trabecular bone surfaces. Bar = 50 μm. *n* = 9 mice for each group. **t** Serum TRACP-5b tested by ELISA. *n* = 5 and 4 mice, respectively. All analyses: unpaired Student’s *t* test. *P < 0.05; **P < 0.01; ***P < 0.001

To determine if inhibition of CXCR4 signaling prevents the accumulation of RCBs in BM, we sorted RCBs from 17-month-old mTmG mice and injected them into 3-month-old NSG mice via tail veins and treated them with vehicle or the FDA-approved CXCR4 inhibitor, plerixafor, once/d for 12 d before sacrifice (Fig. 5a). We found that the plerixafor-treated recipients had significantly fewer tdTomato^+^ RCBs (Fig. 5b, c) and osteoclasts in BM (Fig. S8a) than vehicle-treated recipients. To further examine the effects of plerixafor on RCB accumulation in BM and bone mass of aged mice, we treated 22-month-old WT mice with plerixafor, once/d for 1 month. We found that vehicle- and plerixafor-treated aged mice had similar body weights (Fig. S8b), BM cellularity (Fig. S8c), liver and renal function parameters, including alanine aminotransferase, aspartate aminotransferase and blood urea nitrogen (Fig. S8d, f), LSK pool and subsets (Fig. S8g, h), and neutrophils in BM (Fig. S8i, j) after treatment. However, plerixafor significantly reduced the frequency and numbers of RCBs (RANKL^+^CXCR4^+^B220^hi^IgM^+^) in BM of aged mice (Fig. 5d-g), accompanied by higher trabecular bone volume (Fig. 5h, i) and thickness (Fig. 5j), but similar trabecular number and separation, and cortical thickness (Fig. 5k; Fig. S8k, l), compared to vehicle-treated mice. Consistent with this, plerixafor-treated aged mice had significantly higher mineral apposition (Fig. 5l) and bone formation rates (Fig. 5m), and osteoblast surfaces (Fig. 5n, o) in L1 vertebrae than in vehicle-treated mice. However, serum osteocalcin levels in plerixafor-treated aged mice were similar to those in vehicle-treated aged mice (Fig. 5p). Of note, plerixafor-treated aged mice had significantly lower osteoclast numbers and surfaces (Fig. 5q–s) and TRACP-5b serum levels (Fig. 5t) than vehicle-treated mice. Importantly, we treated osteoblast and osteoclast progenitor cells with increasing doses of plerixafor in vitro and found no significant change in osteoclast- or osteoblast-positive areas, suggesting that plerixafor does not have direct effects on osteoblast or osteoclast formation (Fig. S8m, n).

### The CXCR4 antagonist, plerixafor, prevents RCB accumulation in BM and bone loss in ovariectomized mice

**Fig. 6:**
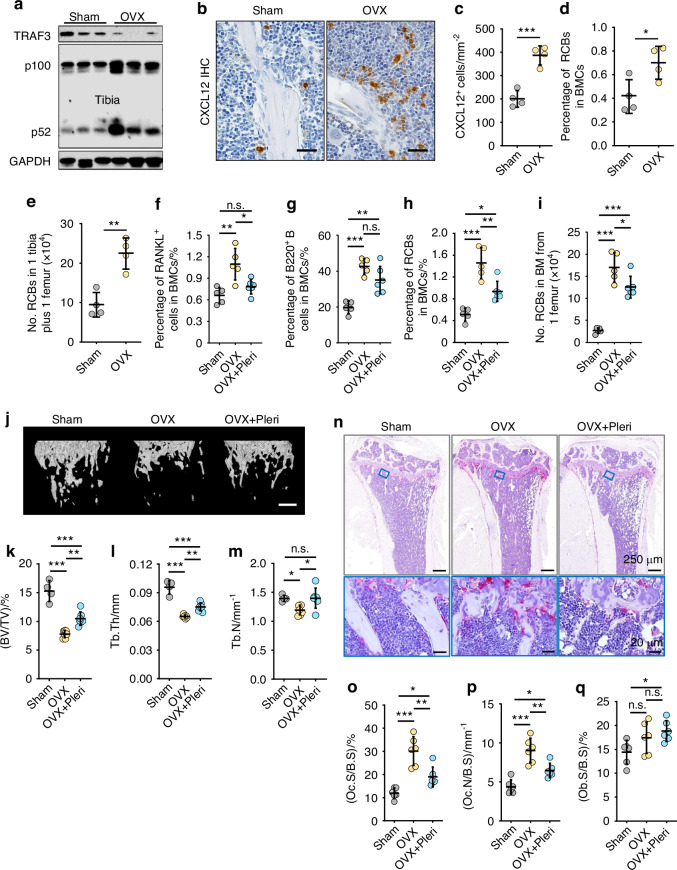
The CXCR4 antagonist, plerixafor, prevents bone loss in ovariectomized mice. **a** TRAF3, NF-κB p100 and p52 and GAPDH levels in bone protein lysates by Western blot. **b** Immunohistochemical expression of CXCL12 in tibial paraffin sections, and **c** numbers of CXCL12^+^ cells. Bar = 25 μm. *n* = 4 mice for each group. **d**, **e** Frequency and numbers of RCBs (RANKL^+^CXCR4^+^B220^hi^IgM^+^CD138^+^) in BM of one femur plus one tibia tested by flow. % RANKL^+^ cells (**f**) and B220^+^ cells (**g**) in total BM cells of sham, vehicle-treated OVX (OVX) and plerixafor-treated OVX (OVX+Pleri) mice. *n* = 5, 5 and 6 mice in these groups, respectively. **h**, **i** Frequency and numbers of RCBs (RANKL^+^CXCR4^+^B220^hi^IgM^+^CD138^+^) in BM cells from sham, OVX and OVX+Pleri mice. **j** μCT 3D reconstruction of femora and values for **k** trabecular bone volume and **l** thickness, and **m** number. Bar = 500 μm. *n* = 4, 7 and 7 mice, respectively. **n** TRAP-stained tibial paraffin sections and **o** osteoclast surfaces and **p** numbers. Bar = 20 μm in lower panel and 250 μm in upper panel. *n* = 6 mice for each group. **q** Osteoblast surfaces counted on H&E-stained tibial paraffin sections. *n* = 6 mice for each group. Analyses: unpaired Student’s *t* test in (**c**–**e**); one-way ANOVA with Tukey’s post-hoc test in all other panels. *P < 0.05; **P < 0.01; ***P < 0.001

Osteoporosis also occurs after the menopause as a result of sex steroid deficiency, and ovariectomy increases bone turnover in mice, resulting in increased TGFβ1 release from resorbed bone.^31^ In addition, ovariectomy causes B cell expansion in BM.^32^ To determine if RCBs are involved in the pathologic bone loss in an estrogen deficiency mouse model, we ovariectomized (OVX) mice, which increased osteoclast numbers in their long bones (Fig. S9a). We found that OVX mice had lower levels of TRAF3 in protein lysates of tibiae than sham mice, associated with increased levels of NF-κB p52 protein (Fig. 6a), numbers of CXCL12-expressing cells (Fig. 6b, c), and frequency and numbers of RCBs in BM (Fig. 6d, e).

We then treated OVX mice with plerixafor to determine if blockade of CXCR4 prevents the accumulation of RCBs in BM and bone loss. We found that the plerixafor-treated OVX mice had significantly fewer RANKL^+^ B cells, but not total B cells, in BM cells than OVX mice treated with vehicle (Fig. 6f, g; Fig. S9b, c), and that they had lower frequency and numbers of RCBs in BM (Fig. 6h, i). These mice also had higher femoral trabecular bone volume, number, thickness, and bone mineral density than vehicle-treated OVX mice (Fig. 6j–m; Fig. S9d–f), associated with significantly fewer osteoclasts (Fig. 6n–p), with no significant changes in osteoblast numbers (Fig. 6q), and a significant increase in adipocyte numbers (Fig. S9g). These findings suggest that ovariectomy-induced TRAF3 degradation and subsequent NF-κB-mediated CXCL12 production facilitated RCB accumulation in BM and that blockade of CXCR4 with plerixafor inhibited RCB accumulation and prevented bone loss in OVX mice.

## Discussion

**Fig. 7:**
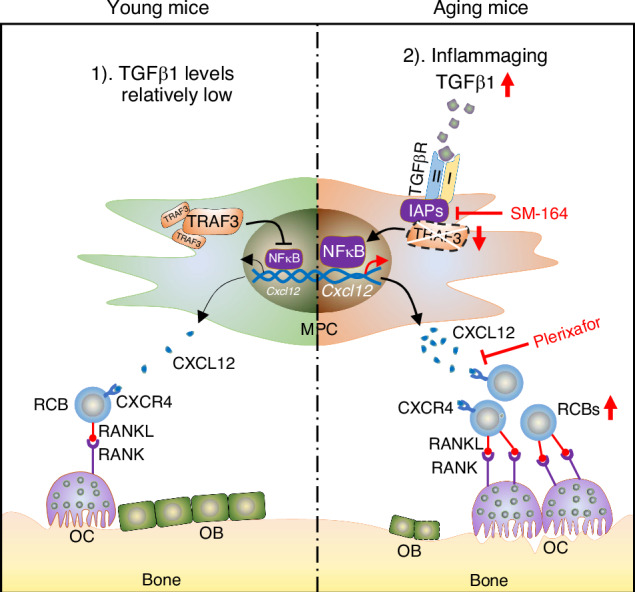
Model showing how TRAF3 reduction in MPCs leads to increased CXCL12 expression by MSCs, with accumulation of RCBs in BM and bone loss during aging. 1) TRAF3 protein levels are optimal in MPCs in young and adult mice to limit NF-κB-dependent *Cxcl12* transcription and CXCL12 levels in BM. Consequently, there are small numbers of RANKL/CXCR4-expressing RCBs in BM and this limits osteoclast formation to maintain bone mass. 2) During aging, levels of active TGFβ1 increase in the bone microenvironment, as we previously reported.^14^ TGFβ binding to its receptors causes ubiquitination and autophagosomal degradation of TRAF3 by the E3 ligases, cIAPs, leading to increased NF-κB (RelA/RelB)-induced expression of the chemokine, CXCL12. RCBs are attracted to and maintained in BM by increased CXCL12 expression by MPCs, leading to enhanced RANKL-induced osteoclastogenesis. SM-164, an IAP inhibitor, can prevent the decrease in TRAF3 protein levels, and the CXCR4 antagonist, plerixafor, can prevent accumulation of RCBs in BM and reduce increased bone resorption during aging

This is the first report of a previously unidentified sub-population of RANKL^+^CXCR4^+^ B cells (RCBs) playing an important role in the pathogenesis of age-related osteoporosis as a result of their numbers increasing in BM in male mice during aging in response to increased expression of CXCL12 by BM mesenchymal progenitor cells (MPCs). Our findings of osteoporosis developing also in adult mice with TRAF3 deleted in MPCs, associated with increased levels of CXCL12, support a model in which TRAF3 expression by MPCs in the BM microenvironment of young and adult mice restricts NF-κB-mediated production of CXCL12, and this limits the numbers of RCBs in BM. We propose that this mechanism restricts bone resorption to optimal levels to maintain skeletal integrity in young and adult mice. As a result of the increased CXCL12, RCB numbers increase in BM during aging, which we propose is through chemoattraction, and cause bone loss by stimulating excessive resorption (Fig. 7). Several novel findings support this mechanism: 1) RANKL protein levels are much higher in murine BM cells than in bone or BM plasma; 2) injection of RCBs from aged mice caused more bone loss in young NSG mice than the same number of RCBs from young mice; 3) more RCBs from aged mice were attracted toward WT MPCs than RCBs from young mice in vitro, and more of these RCBs were attracted towards Prx1^Cre^;TRAF3^fl/fl^ MPCs, each associated with increased expression of CXCL12 by the MPCs. This increase in RCB migration was prevented by pre-treatment of RCBs with the CXCR4 antagonist, plerixafor; 4) treatment of aged mice with the IAP inhibitor, SM-164, for 1 month not only prevented TRAF3 degradation, but also reduced RCB numbers in BM and increased bone mass; and 5) treatment of aged mice with plerixafor for 1 month not only reduced RCB numbers in BM, but also increased bone mass.

Osteoblastic cells are a critical source of RANKL for osteoclast formation, given that these cells are on or close to bone surfaces and are located adjacent to osteoclasts and osteoclast precursors. Although we found that B220^hi^ B cells with high expression of RANKL protein on their cell membranes comprise only ~2% of B cells in BM, they are present in clusters adjacent to bone surfaces and, importantly, in much higher numbers than RANKL^+^ MPCs, suggesting that they are a more important source of RANKL during aging. However, establishing the relative importance of these various cell types for osteoclastogenesis will require further investigation.

Onal et al. reported that mice with RANKL deletion in B lymphocytes are only partially protected from OVX-induced bone loss.^33^ However, these mice also have defects in development and function of B lymphocytes due to cKO of RANKL, which would likely have affected a broad heterogenous spectrum of B cell subtypes, including a reduction in Pre-B cells.^33^ These defects may have attenuated the protective effect of loss of RANKL in B cells on bone in this cKO mouse model, given that RANKL is required for B cell and lymph node development^34^ and B cells are also a critical cellular source of OPG.^35^ A significant limitation of our study is that we have not generated or used these RANKL cKO mice to demonstrate conclusively that they are responsible for the bone loss that occurs during aging. However, the O’Brien group, which generated these mice, has not maintained or aged these mice (personal communication). Thus, to carry out this experiment optimally, we would also have to generate mice deficient in both RANKL and CXCR4, which is beyond the scope of this study. In addition, Engdahl, et al. reported that the bone loss caused by estrogen deficiency or arthritis was not prevented in μMT-deficient mice.^36^ However, compared to WT mice, total B cells in BM of μMT-deficient mice were reduced by over 80% and immature B cells (B220^low^IgM^+^) and plasma cells (CD138^+^TACI^+^) are reduced by over 90%, while serum IgG and IgM levels are reduced to an almost undetectable level,^36^ indicative of profound effects on B cells in these mice. Our study identified surface markers of a specific RANKL-expressing B cell subset, raising the possibility of precisely targeting this CXCR4-expressing subset to protect against bone loss in estrogen deficiency- and age-related bone loss.

Osteocytes are an important source of RANKL for bone remodeling in adult mice^37^ and comprise 90%-95% of cells in bone tissue. They also become senescent during aging, associated with increased expression of pro-inflammatory cytokines, which can promote increased RANKL production.^38^ However, our data suggest that osteocyte-produced RANKL may not be so important during aging. For example, RANKL levels are similar in bone from young and old mice (Fig. 1a). One possible explanation for this could be that osteocyte numbers decrease during aging^39^ along with the reduction in bone mass, and this could offset the effects of their increased expression of cytokines. RANKL is expressed also by various types of cells in BM,^40^ including B and T lymphocytes, osteoblastic cells, and MPCs, but, with the exception of B cells, all of these are present in very small numbers in BM (Fig. 1b).

We have no direct evidence to definitively define the origin of RCBs, but IgM and IgD co-expression and high expression of B220 suggest that they are not immature B cells, which typically are B220^low^IgM^high^. High expression of IgM and IgD and of CD138 by RCBs suggests that they are plasmacytoid^41^ and/or transitional B cells.^42^ Pre/Pro-B cells develop in BM and mature functionally in secondary lymphoid organs^43^; they give rise to age-associated B cells (ABCs) whose numbers are increased in BM of aged mice, but we found that few RCBs express CD11c, T-bet, or CD80, key markers of ABCs.^25,26^ RCBs are more likely to be mature naïve B cells because they are IgD^high^; however, they have low-to-intermediate expression of CD138, suggesting they may not be naïve and could be mature follicular B cells. Kim et al. reported that low-level expression of CD138 marks natural anergic B cells, but not plasma cells, in spleen,^44^ suggesting the possibility that RCBs originate in the spleen and enter the BM in increased numbers during aging. CD138 expression by RCBs is surprising; however, it is expressed not only by plasma cells, but also by numerous normal and neoplastic epithelial cells and sarcomas where it has diverse effects on cell growth and prognosis.^45^ Thus, its function in RCBs could be related to growth of these cells, but study of that is outwith the scope of this paper.

We posit that RCBs are attracted to the BM, rather than being retained there by CXCR4 because donor-derived tdTomato^+^ RCBs transferred via tail vein accumulated in BM of NSG recipient mice, and this accumulation was prevented by plerixafor. In addition, intraperitoneal injection of plerixafor significantly reduced RCB numbers in BM of aged mice, but it did not change hematopoietic stem/progenitor cells or neutrophils, suggesting that unlike HSCs being retained in BM, RCBs may reenter the BM during aging. Additional definitive genetic studies will be required to determine if RCBs are attracted in increased numbers to BM by CXCL12 in vivo during aging, if they have increased proliferative capacity, reduced apoptosis, any additional functions, what their fate is, and if they are senescent.

The RANKL mAb, denosumab, efficiently inhibits bone resorption in patients with common skeletal diseases, including menopause and age-related osteoporosis.^46^ However, given RANKL’s role in B cell and lymph node development,^47^ denosumab could impair immune responses. Indeed, infections were more common in denosumab- than placebo-treated postmenopausal women in a clinical trial,^48^ highlighting the challenge of optimizing inhibition of osteoclastogenesis and maintaining immune responses.^49^ Our findings that RCB numbers are increased in OVX mice and that plerixafor prevented this increase and ovariectomy-related bone loss in female mice suggest that reduction of RCB numbers in BM may be an attractive alternative approach for treatment of postmenopausal osteoporosis. This is particularly relevant given the reluctance of many patients to take and of some physicians to prescribe the effective anti-resorptive drugs, bisphosphonates^50^ and denosumab,^50^ because of rare side-effects, including atypical femoral fractures and osteonecrosis of the jaw,^51^ and the fact that treatment with current FDA-approved drugs that stimulate new bone formation is limited to 2 years.^52^ Indeed, SM-164 or plerixafor reduced RCB numbers in BM, and importantly, they also increased bone volume in 22-month-old mice by inhibiting bone resorption *and* stimulating bone formation. Denosumab^46^ and bisphosphonates,^53^ in contrast, do not stimulate new bone formation.^54^

SM-164, was developed to kill cancer cells by degrading both cIAP (cellular inhibitor of apoptosis) and XIAP (X-linked inhibitor of apoptosis) proteins.^28,29^ In an vivo mouse model, it also eliminated early-stage metastatic breast cancer and significantly reduced the progression of advanced bone and lung metastases from the triple-negative human breast cancer cell line, MDA-MB-231, in a TNF-dependent mechanism.^55^ We did not observe any serious side-effects in the mice we treated with SM-164 for up to 4 weeks in that^55^ or the current study, and the mice had normal body weights at the end of the experiments. Our findings suggest that SM-164 might be useful in the prevention and treatment of age-related osteoporosis, but further studies will be required to determine if it is efficacious in longer-term studies in aging mice and if it has serious side effects in this setting.

Plerixafor is FDA-approved to treat HIV-infected patients^56^ and to release HSCs from BM into the blood before autologous stem cell transplantation for hematologic malignancies.^57^ In this study, we found that plerixafor significantly increased bone volume and prevented RCB accumulation in BM of aged mice. We did not find any significant changes in numbers of hematopoietic stem/progenitor cells (HSPCs) or neutrophils in BM of aged mice following plerixafor administration, or in osteoblastic and osteoclastic differentiation from their progenitor cells after treatment with plerixafor in vitro. In addition, plerixafor prevented RCBs from being recruited to BM, but minimally changed the HSPC population that can be mobilized from BM. The significant increase in CXCL12 protein levels in BM in aging mice may provide a stronger chemoattractant force to maintain HSPCs in BM, which may explain the difference between recruitment and mobilization upon CXCR4 blockade. The failure of plerixafor to significantly affect the number of hematopoietic cells, especially neutrophils, in BM of these aging mice appears surprising because they also express CXCR4, and plerixafor could enhance their egress from the BM of aging mice. However, plerixafor was absorbed and reached peak plasma concentrations rapidly (occurring in 0.5 h) after subcutaneous injection, cleared rapidly with a median terminal half-life of 4.6 h, and maintained substantially higher plasma concentrations than those achieved in BM.^58^ This suggests that cells expressing CXCR4^+^ in the circulation, including RCBs, are possibly exposed to effective antagonism earlier and at higher levels than their counterparts within marrow niches. This differential exposure could shift the net effect from promoting egress to impairing homing, consistent with our data showing that plerixafor blocked RCB-cell chemotaxis toward MSCs that have high CXCL12 expression (Fig. 3q, r) and reduced their BM homing in recipient mice (Fig. 5a, b). A phase I study indicates that CXCR4^+^ blasts and lymphocytes significantly increased in the peripheral blood following plerixafor administration, while numbers of these cells in BM were not decreased.^59^ Under the experimental conditions in this study, the dominant outcome appears to be inhibition of homing rather than induction of mobilization. In addition, plerixafor could have off-target effects to explain the positive effects on bone volume. For example, it is an allosteric agonist of CXCR7,^60^ which is another receptor for CXCL12, but it functions differently from the primary receptor, CXCR4, acting more as a scavenger to internalize and degrade CXCL12, thereby regulating its availability for CXCR4.^60^ CXCR7 activation could perturb an integrated CXCL12/CXCR4/CXCR7 signaling network and thereby alter cell trafficking in a non-canonical manner. Whether CXCR7 plays a contributory role in this setting to explain our findings will require further investigation. While it is hard to fully exclude effects of plerixafor on other types of BM cells, such as monocytes, macrophages and T cells, on the increase in bone mass in plerixafor-treated aged mice, Manologas et al. reported that cKO mice with *Cxcl12* specific deletion in mesenchymal lineage cells (Prrx1-Cre-driven) had significantly higher bone mass after OVX, associated with significantly fewer B cells in BM.^61^ In line with our findings, the CXCL12/CXCR4 axis could be a therapeutic target for age- and OVX-related bone loss. We did not detect obvious adverse effects in the mice treated with plerixafor for 4 weeks. However, further studies will be required to determine if it can be administered longer-term to mice to prevent age-related bone loss since it has tolerable, but sometimes severe side effects that may be acceptable in patients with malignancies and HIV infections,^62,63^ but not in patients with benign diseases, such as osteoporosis.

Limitations of our study include the following: 1) CXCR4 is expressed by multiple cell types, and we cannot exclude the possibility that the bone anabolic effect of plerixafor is mediated by cells other than RCBs; 2) similarly, CXCL12 is produced not only by MPCs, but also by other cell types. It is likely that during aging, increasing amounts of CXCL12 are produced by multiple cell types in BM, thereby attracting RCBs; 3) CD138 is a marker of plasma cells. The importance of plasma cell-produced RANKL in periarticular bone loss has been reported in patients with rheumatoid arthritis.^64^ Whether RCBs are the same as those RANKL-expressing plasma cells discovered in rheumatoid arthritis or if they represent a new sub-cluster of age-related B cells needs to be studied further; and 4) we have not demonstrated that mice deficient in CXCR4 in their B cells do not have accumulation of RCBs in their BM and are resistant to age-associated bone loss.

## Methods

### Animals

Mice with TRAF3 conditionally knocked out in mesenchymal, including osteoblast, lineage cells were generated by crossing TRAF3^f/f^ mice from Dr. G. A. Bishop in the University of Lowa with Prx1^Cre^ mice (Jackson Lab, Stock #: 005584) to generate TRAF3^f/f^Prx1^Cre^ mice, which we call P-cKO mice. P-cKO mice and their WT (TRAF3^f/f^) littermates were sacrificed at 12 months of age. Mice with TGFβRII conditionally knocked out in mesenchymal lineage cells were generated by crossing TGFβRII^f/f^ mice (Jackson Lab, Stock #: 012603) with Prx1^Cre^ mice. TGFβRII^f/f^Prx1^Cre^ mice (which we call T-cKO mice) and their WT (TGFβRII^f/f^) littermates were sacrificed at 15 months of age. ROSA^mT/mG^ mice (Jackson Lab #007676) have cell membrane-localized tdTomato (mT) fluorescence expressed widely in cells/tissues prior to Cre recombination. All these mouse lines are in a C57B6 background. Male NSG mice (NOD-Prkdc^scid^IL2rγ^em1Smoc^; #NM-NSG-001) aged 8 weeks were purchased from Shanghai Model Organisms Center, Inc. WT C57B6 mice were purchased at breeding age from the National Cancer Institute (Frederick, MD, USA) and bred to generate young WT mice that were sacrificed at 2-3 months of age. Aged C57B6 mice (18-22-month-old) were provided by the National Institute on Aging (Bethesda, MD, USA). Aged mice were injected intraperitoneally with vehicle, plerixafor (5 mg/kg/d)^65^ or SM-164 (3 mg/kg/d)^66^ once/d for one month and sacrificed for further analysis. 12-month-old male WT and P-cKO mice were injected with vehicle or SM-164 (3 mg/kg/d) once/d for one month before sacrifice. 3-month-old female C57B6 mice were ovariectomized (OVX), as previously described,^67^ and treated with vehicle or plerixafor (5 mg/kg/d)^65^ once/d for one month and sacrificed for further analysis. Mice were randomized and grouped according to body weight for study. All animal procedures were conducted in compliance with all applicable ethical regulations using procedures approved by the University of Rochester Committee for Animal Resources.

### Reagents

The following Abs were purchased from Santa Cruz Biotechnology Inc.: anti-TRAF3 (Cata #: sc-947), -Ubiquitin (Cata #: sc-8017) and -GAPDH (Cata #: sc-32233). Abs against human/mouse cIAP (Cata #: MAB3400), human/mouse XIAP (Cata #: MAB822) and human/mouse CXCL12/SDF-1 (Cata #: MAB350-100) were purchased from R&D. These primary Abs were used at the following concentrations: Ubiquitin (1:200) and all others (1:500). β-actin Ab (Cata #: A5441) was purchased from Sigma-Aldrich and used at a concentration of 1:5 000.

The following primary and secondary Abs were used for flow cytometry: PE-conjugated anti-CD4 (eBioscience; Cata #: 12-0041-81), APC-conjugated anti-CD19 (eBioscience; Cata #: 17-0193-82), PE-Cy7-conjugated anti-B220 (Biolegend; Cata #: 103222), PE-Texas red-conjugated streptavidin (BD; Cata #: 551487), biotin-conjugated anti-Lineage (BD; CD3e, Cata #:51-01082J; B220, Cata #: 51-01122J; Ter119, Cata #: 51-09082J; CD11b, Cata #: 51-01712J), FITC-conjugated anti-CD45 (eBioscience; Cata #: 11-0451-82), APC-conjugated anti-CD45 (eBioscience; Cata #: 17-0454-82), PE-conjugated anti-Sca1 (eBioscience; Cata #: 12-5981-81), PE-Cy7 conjugated anti-Sca1 (eBioscience; Cata #: 25-5981-81), PE-conjugated anti-CD51 (eBioscience; Cata #: 12-0512-81), biotin-conjugated anti-RANKL (eBioscience; Cata #: 13-5952-82), APC-conjugated anti-IgD (eBioscience; Cata #: 17-5993-82), FITC-conjugated anti-IgM (eBioscience; Cata #: 11-5790-85), PE-conjugated anti-CD138 (Invitrogen; Cata #: 300506), BV605-conjugated anti-CXCR4 (Biolegend; Cata #: 146519), PE-Cy7-conjugated anti-CD3e (eBioscience; Cata #: 25-0031-81), BV785-conjugated anti-CD11c (Biolegend; Cata #: 117336), Percp-Cy5.5-conjugated anti-CD80 (BD; Cata #: 560526), BV711-conjugated anti-T-bet (Biolegend; Cata #: 644820), APC-R700 conjugated Streptavidin (BD; Cata #: 565144). Primary and secondary Abs for Flow were used at 1:100 dilution, and PE-conjugated anti-CD138 was used at 1:33 dilution.

The following Abs were used for immunofluorescence staining: primary Abs: anti-mouse RANKL polyclonal Ab (Proteintech; Cata #: 23408-1-AP), anti-mouse Nestin monoclonal Ab (Beyotime; Cata #: AN205) and anti-mouse CD45R (B220) monoclonal Ab (eBioscience; Cata #: 14-0452-82) with 1:100 dilution. Secondary Abs: Alexa Flour 488/568-conjugated secondary Abs (Invitrogen; Cata #: A-21042/A-21124) and Rat AF647 (Invitrogen; Cata #: A-21247) with 1:500 dilution.

Recombinant murine TGFβ1 (#7666-MB) was from R&D Systems. MG132 (Cata #: M8699) and chloroquine (Cata #: C6628) were from Sigma-Aldrich. ELISA kits for osteocalcin (Cata #: LS-F22474) were from LifeSpan BioScience, Inc., for TRACP5b (Cata #: MBS763504) from MyBioSource, Inc. and for mouse TRANCE/RANKL (Cata #: EMTNFSF11) from Invitrogen. Plerixafor (Cata #: HY-10046) and SM-164 (Cata #: HY-15989) were from MedChemExpress.

### Ex vivo/in vitro ubiquitination assays

4h prior to sacrifice, 3- and 18-month-old C57B6 mice were intraperitoneally injected with a single dose of MG132 (15 mg/kg body weight) and of chloroquine (CQ, 60 mg/kg body weight). Protein was harvested separately from cortical bone and BM. CD11b^+^ leukocytes were magnetically isolated from BM. Protein lysates from total BM cells and sorted cells were processed further to determine the levels of ubiquitinated TRAF3 protein. For in vitro assays, 1.5 × 10^7^ primary BM cells were re-suspended in 8 mL OCP culture medium (10% FBS in α-MEM containing 1% non-essential amino acids (NEAA), 1% pen/Strep, 1% glutamine and 2% M-CSF) and seeded in 100 mm dishes. 2 d later, cells suspended in culture medium were discarded and the attached cells were treated with CQ (100 μmol/L) or MG132 (20 μmol/L) for 4 h before treatment with vehicle or RANKL (10 ng/mL) plus CQ or MG132 for 8 h. These cells were lysed in RIPA lysis buffer containing 20 mmol/L HEPES, 250 mmol/L NaCl, 20 mmol/L Tris–HCl, 0.5% NP-40, 2 mmol/L EDTA, 2 μL leupeptin, 2 μg/mL aprotinin, 1 mmol/L DTT, 1 mmol/L PMSF, 1 tablet (in 10 mL lysis buffer) of Protease Inhibitor Cocktail (PIC; Roche, Cata #: 04693159001), 1 mmol/L N-ethylmaleimide (Sigma, Cata #: E3876) and 1 µg/m Lubiquitin aldehyde (Enzo Life Sciences, Cata #: BML-UW8450-0050) to limit deubiquitination. 300–500 μg whole cell protein lysates were incubated with anti-TRAF3 Ab, and precipitated proteins were subjected to WB analysis using anti-Ub Ab.

### Enzyme-linked immunosorbent assay (ELISA)

Serum levels of osteocalcin and TRACP5b, and levels of RANKL in protein lysates of bone, BM and BM plasma were tested by ELISA according to the manufacturer’s instructions. For sample preparation, BM cells were flushed out from 1 tibia and 1 femur from each mouse with 250 μL PBS containing protease inhibitor cocktail (PIC; Roche, Cata #: 04693159001; 1 tablet in 10 mL PBS). BM cells were spun down immediately, and supernatant containing intercellular fluid was collected and called BM plasma. Cortical bone was chopped and homogenized and immediately lysed in 250 μL T-per lysis buffer containing PIC. BM cells were also lysed in 250 μL T-per with PIC. Bone, BM, and BM plasma proteins were lysed in equal volumes of protein lysis buffer, and equal volumes of these lysates were added to each well of pre-coated plates for incubation. ELISA plates were analyzed by investigators blinded to sample identity by reading absorbance using a microplate reader set to 450 nm with wavelength correction set to 540 nm.

### Flow cytometry

BM was flushed out with FACS buffer (2% FBS in PBS) from leg bones dissected from young and aged C57B6 mice that had been treated with vehicle or plerixafor, as well as TβRII-cKO and P-cKO mice and their respective WT littermates that had been treated with vehicle or SM-164. After red blood cell lysis, BM cells were counted and 1 × 10^7^ cells/test were incubated with a primary Ab staining panel, including 1 μL anti-IgM-FITC, anti-RANKL-biotin, anti-B220-PE-Cy7, anti-IgD-APC, anti-CD80-Percp-Cy5.5, anti-CXCR4-BV605, anti-CD11c-BV785 and 3 μL anti-CD138-PE in 100 μL FACS buffer at 4 °C for 30 min, followed by incubation with 1 μL Texas Red- or APC-R700-conjugated streptavidin in 100 μL FACS buffer at 4 °C for 20 min for detection of biotin-conjugated RANKL Ab for RANKL^+^ B cell measurements. To test for intracellular expression of T-bet, BM cells stained with anti-surface antigen Abs were fixed and permeabilized using BD Cytofix/Cytoperm solution kit (BD, Cata #: 51-2090/2091KZ), followed by incubation with 3 μL anti-T-bet-BV711 in 100 μL Perm/Wash buffer at 4 °C for 30 min. After two washes using BD Perm/Wash buffer and FACS buffer consecutively, these cells were ready for FACS analysis. To collect bone-associated cells, bones with BM previously flushed out were cut into <1 mm fragments and digested twice in collagenase type I (Stem cell technologies, Cata #: 7902) for 30 min and then 60 min. Cells were washed and filtered through a 40 μm pore size cell strainer. To test various types of cells for RANKL expression, 1 × 10^7^ BM cells or 1 × 10^6^ bone-associated cells were stained with primary Abs, including anti-CD19-APC, anti-B220-PE-Cy7 and anti-RANKL-biotin, anti-Lineage-PE-Cy5, anti-CD45-FITC, anti-Sca1-PE-Cy7 and anti-CD51-PE or anti-CD4 PE in 100 μL FACS buffer at 4 °C for 30 min, and then followed by streptavidin-conjugated secondary Ab incubation. After one wash with FACS buffer, these stained cells were acquired using a flow cytometer (FACS LSR II; BD Biosciences). We first gated BM cells using a “J”-shape gate and excluded DAPI-positive dead cells and then used forward scatter height (FSC-H) versus forward scatter area (FSC-A) plot, and side scatter height (SSC-H) vs side scatter area (SSC-A) plot to exclude doublets. Expression of cell surface and intracellular markers were then analyzed. These gating strategies were applied in all flow analyses in this study. FlowJo software was used for data analysis.

### Cell sorting by FACS and magnetic columns

To sort B cell subpopulations, (7–8)× 10^7^ BM cells from 2 to 3-month-old and 22 to 24-month-old C57B6 mice were stained with biotinylated anti-CD11b Ab (Miltenyi, Cata #: 130-109-284; 5 μL/10^7^ cells) at 4 °C for 30 min, followed by anti-biotin MicroBeads (Miltenyi, Cata #: 130-090-485; 3 μL/10^7^ cells) at 4 °C for 20 min and passed through a LS column. B lymphocytes were thus enriched in the negatively-selected cells. These negative cells were counted and then incubated with 2 μL (per 10^7^ cells) of anti-B220, -IgM, -IgD, -CD138 and -CXCR4 Abs at 4 °C for 30 min. After being washed twice with 0.5% BSA-PBS and re-suspended in FACS buffer at a concentration of 2 × 10^7^ cells/mL, the targeted cell populations were sorted by FACS for various purposes, including RNA extraction, culture and cytospinning using BD FACSAria II sorting equipment.

Magnetic sorting was also used to isolate CD45^-^ or CD11b^+^ cells from mouse BM. Briefly, 8 × 10^7^ BM cells were incubated with 5 μL (per 10^7^ cells) biotinylated anti-CD45 (Miltenyi, Cata #: 130-101-952), or 5 μL (per 10^7^ cells) anti-CD11b Ab (Miltenyi, Cata #: 130-109-284) at 4 °C for 30 min, followed by 3 μL (per 10^7^ cells) anti-biotin MicroBeads (Miltenyi, Cata #: 130-090-485) at 4 °C for 20 min. After being washed twice with 0.5% BSA-PBS, each sample was sorted using LS columns. Isolated CD45^-^ cells and CD11b^+^ cells were lysed in RIPA for RNA extraction in anti-deubiquitination protein lysis buffer for TRAF3 ubiquitination analysis.

### NSG mice injected with sorted RANKL^+^B220^hi^IgM^+^ B cells

To isolate RANKL^+^B220^hi^IgM^+^ B cells (RCBs), 3- and 20-month-old ROSA^mTmG^ male mice were sacrificed, and BM single-cell suspensions were prepared for further staining. RANKL-expressing BM cells were first enriched through positive selection using a magnetic isolation system with biotin-conjugated anti-RANKL Ab as primary and streptavidin-conjugated MicroBeads as secondary labeling. RANKL^+^B220^hi^IgM^+^ B cells were further flow-sorted from the positively selected RANKL-expressing BM cells. 5 × 10^5 ^RCBs from young or aged ROSA^mTmG^ male mice were injected into 3-month-old male NSG mice via tail vein. Twelve days later, the recipient mice were sacrificed for bone phenotype analysis.

To isolate RANKL^+^ B cells (R^+^BCs; RANKL^+^B220^hi^IgM^+^) and RANKL^-^ B cells (R^-^BCs; RANKL^-^B220^hi^IgM^+^) from BM of aged mice, RANKL^+^ cells were enriched through positive selection using a magnetic isolation system, as above. R^+^BCs and R^-^BCs were further flow-sorted from the positively and negatively selected RANKL^+^ and RANKL^-^ BM cells, respectively. 2 × 10^5^ R^+^BCs or R^-^BCs were injected into each 3-month-old male NSG mouse via tail vein. Twelve days later, the recipient mice were sacrificed for bone phenotype analysis.

To isolate B220^+^ B cells, 12-month-old male WT and P-cKO mice were sacrificed, and BM single-cell suspensions were stained with biotin-conjugated anti-B220 Ab as primary and streptavidin-conjugated MicroBeads as secondary labeling, and then positively selected using a magnetic isolation system. 8 × 10^5^ sorted B220^+^ B cells from WT or P-cKO mice were injected into the 3-month-old male NSG mice via tail vein. Twelve days later, the recipient mice were sacrificed for bone phenotype analysis.

### Co-culture of immune cells with osteoblast and osteoclast precursors

2.5 × 10^4^ BM cells from 1-month-old WT mice were seeded in each well of 96-well plates with OCP culture medium (α-MEM with 10% FBS, 1% pen/strep, 1% NEAA and 2% M-CSF) for 2 d. CXCR4^+^B220^++(hi)^IgM^+^IgD^+^ CD138^+^ B cells were sorted from BM cells from 2-month-old young and 22-month-old aged male mice using FACS, and pretreated with RPMI 1640 medium with 10% FBS, 1% pen/strep, 5 μg/mL LPS, 20 ng/mL PMA and 2.5 μg/mL anti-CD40 Ab for 48 h in curved bottom 96-well plates. For co-culture, the unattached cells in OCP culture wells were gently aspirated and suspended with 3 × 10^4^ pre-treated B cells in 200 μL OC/B cell culture medium (RPM I1640 medium with 10% FBS, 1% pen/strep, 2% M-CSF, 1% NEAA, 20 ng/mL phorbol 12-myristate 13-acetate (PMA; Calbiochem) and 2.5 μg/mL anti-CD40 Ab) and added into the OCP-coated wells and maintained for 2 d. In the supernatant-treated group, 100 μL of supernatant from pre-treated (for 48 h) B cells were mixed with 100 μL of the osteoclast/B cell culture medium mentioned above (but containing 4% M-CSF, 2% NEAA), and added into each OCP-coated well and maintained for 2 d. Cell culture was terminated by fixation with 10% formalin. TRAP staining was performed for measurement of the area of TRAP^+^ cells.

### Micro-CT and bone histomorphometric analysis

For in vivo assessment of bone formation, mice were given injections of calcein (10 mg/kg) 5 and 1d before sacrifice, according to our standard protocol.^14^ Right tibiae and T12–L1 or L2-L3 vertebrae were fixed in 10% neutral buffered formalin for 2 d. Micro-CT scanning with a resolution of 10.5 µm was performed using a vivaCT 40 instrument (Scanco Medical), and trabecular bone parameters were assessed in a region 1–4 mm from the edge of growth plates in femoral metaphyses and in the entire trabecular bone area of the first lumbar vertebrae, as we described previously.^30^

Bone specimens were then processed sequentially through two changes of 95% ethanol for 1 h each, two changes of 100% ethanol for 1 h each under vacuum conditions, two changes of LR white hydrophilic medium (Polyscience; Cat #: 17411-500) for 1 h each without vacuum suction, two changes of LR white hydrophilic medium for 12 h each under vacuum conditions, and then changed into fresh LR white hydrophilic medium and heated at 60 °C overnight to cure the plastic. 10 d later, 4 µm-thick sections were cut from the plastic blocks using a Shandon Finesse ME microtome. Calcein double labeling was assessed in unstained slides under fluorescence microscopy, and dynamic parameters of bone formation were measured to allow calculation of mineral apposition rate (MAR) and bone formation rate (BFR).^14,68^ For von Kossa staining, 4 µm-thick vertebral coronal plastic sections were stained using 1% aqueous silver nitrate solution placed under ultraviolet light for 20 min.

Left tibiae and L2- or L3–L5 vertebrae were fixed in 10% neutral buffered formalin for 2 d, maintained in 70% ethanol for 2 d, decalcified in 10% EDTA for 14d and then embedded in paraffin. Static parameters of bone resorption and formation were blindly assessed in 4 µm-thick H&E- and TRAP-stained sections by an investigator who was not involved in the sample collection and group assignment using an OsteoMeasure Image Analysis System (Osteometrics, Decatur, GA).

### Western blotting

Mouse long bones were homogenized and lysed with T-Per lysis buffer containing PIC (Roche, Cata #: 11697498001). BM cells and cultured cells treated with various reagents were lysed in PIC-containing RIPA lysis buffer (Millipore, Cata #: 20-188). After incubation at 4 °C for 30 min, the protein lysates were centrifuged at 16.2 × 10^3^ *g* for 15 min and the supernatant was collected. These protein lysates were boiled with 4 × loading buffer and adjusted to a final protein concentration of 1 μg/μL. 20 μg of protein lysates or all proteins pulled down by anti-TRAF3 Ab from 300 μg and 500 μg total protein samples were loaded in 10% SDS-PAGE gels and transferred onto polyvinylidene difluoride (PVDF) membranes. Membranes were incubated with the primary Ab overnight at 4 °C and then with horseradish peroxidase-linked secondary Ab (Bio-Rad) for 2 h at room temperature. The membranes were incubated with ECL substrate, and targeted proteins were detected using an imaging system from Bio-Rad. Densitometric analysis was performed using the Bio-Rad Image Lab 5.1 software.

### Immunofluorescence staining

For immunostaining, 10-μm-thick cryosections from tibiae that had been decalcified with 10% EDTA for 21 days were incubated with 10% normal goat serum in phosphate-buffered saline with 0.05% Tween-20 for 30 min at room temperature to minimize non-specific binding. Primary anti-mouse RANKL, Nestin, and B220 Abs were diluted 1:100 and incubated with the cryosections overnight at 4 °C. On the 2nd day, the sections were incubated with Alexa Fluor 488/568-conjugated secondary Abs for 1 h at room temperature. Following staining, the slides were mounted with Vectashield medium containing DAPI (Vector Laboratories; Cata #: H-1200) for nuclear counterstaining. For cell staining, BM cells freshly harvested from 22-month-old (aged) C57B6 mice were suspended into single-cell suspensions and depleted of red blood cells before staining. 1 × 10^7^ cells were incubated with PE-conjugated anti-B220 Ab and biotin-conjugated anti-RANKL Ab in 100 μL FACS buffer at 4 °C for 30 min. To detect intracellular RANKL expression, the stained cells were fixed and permeabilized using a BD Cytofix/Cytoperm solution kit (BD, Cata #: 51-2090/2091KZ) and then incubated with biotin-conjugated anti-RANKL Ab for 1 h at room temperature, followed by incubation with FITC-conjugated streptavidin in 100 μL FACS buffer at 4 °C for 30 min for detection of biotin-conjugated RANKL Ab in the cytosol and on the cell membrane. After 2 washes using BD Perm/Wash buffer and FACS buffer consecutively, the cells were co-stained with DAPI for nuclear visualization. The stained cells were then pelleted by centrifugation and resuspended, and a drop of the cell suspension was applied to glass slides. A coverslip was then carefully placed over the cells, and the prepared slides were stored in the dark at 4 °C until imaging with a Zeiss Axio Observer A1 inverted microscope. Images were analyzed using ZEN software (version 3.4.91.00000; blue edition; Carl Zeiss Microscopy GmbH).

### Quantitative real-time PCR

1 μg RNA extracted from sorted/cultured murine cells and human vertebral specimens was reverse-transcribed to cDNA using an iSCRIPT cDNA Synthesis kit (Bio-Rad). The expression levels of *Rankl, Opg, Cxcl12* and the entire set of chemokine genes was measured using an iCycler RT qPCR machine (Bio-Rad), which detected the level of fluorescence signals emitted as iQ SYBR SuperMix (Bio-Rad) binding to dsDNA during DNA extension. Primer sequences of mouse genes are as follows: *Tnfsf11 (Rankl)*, forward, 5′-CAGAAGGAACTGCAACACAT-3′, and reverse, 5′-CAGAGTGACTTTATGGGAACC-3′; *Tnfrsf11b* (*Opg)*, forward, 5’-TACCTGGAGATCGAATTCTGCTT-3’, and reverse, 5’-CCATCTGGACATTTTTTGCAAA-3’; *Cxcl12*, forward, 5’-GCATCAGTGACGGTAAAC-3’, and reverse, 5’-GCAGCCTTTCTCTTCTTC-3’; *Gapdh*, forward, 5′-GGTCGGTGTGAACGGATTTG-3′, and reverse, 5′-ATGAGCCCTTCCACAATG-3′. Primer sequences of human genes are as follows: *Cxcl12*, forward, 5’-ATGAACGCCAAGGTCGTG-3’, and reverse, 5’-CTTTAGCTTCGGGTCAATGC-3’; *Gapdh*, forward, 5’-AAGGTGAAGGTCGGAGTCAAC-3’, and reverse, 5’-GGGGTCATTGATGGCAACAATA-3’. The relative abundance (ΔCT) of each gene was calculated by subtracting the GAPDH CT value from the corresponding CT value of specific genes, and ΔΔCT values were obtained by subtracting the ΔCT values of control samples from the others, and then raised to the power 2 (2^−ΔΔCT^) to yield fold changes relative to the controls. The relative expression levels of chemokine genes in CD45^-^ BM cells from 9-month-old P-cKO and 24-month-old WT mice were compared with those from 9-month-old WT mice. A heat map of chemokine gene expression was generated using a heatmap function natively provided in R, a language and environment for statistical computing developed by R Core Team.

### Human specimen collection

We followed a protocol with informed consent from all patients or their guardians that was approved by the Research Subjects Review Board of the University of Rochester Medical Center. These human studies were performed with adherence to the relevant ethical regulations (Declaration of Helsinki). We collected samples of vertebral bone that were removed from pediatric and adult patients undergoing elective surgery to correct spinal scoliosis and degenerative conditions, including cervical spondylosis, lumbar spinal stenosis, and disc herniation. A Rongeur was used to remove portions of the spinous processes in posterior cervical, thoracic, and lumbar spine procedures. In anterior cervical spine procedures, a Kerrison Rongeur was used to remove bony portions of the anterior overhang of the cervical vertebrae. These bone samples would typically have been discarded as part of the surgical procedure. The study enrolled 55 subjects, including 28 females and 27 males, ranging from 8- to 87-year-old, of which 26 subjects were 8–18-year-old children (10 males, 16 females) and the remaining 29 were middle-aged to elderly from 53 to 87 years (18 males, 11 females). Subjects with tumors, active systemic, immunologic, inflammatory, or metabolic disorders that might affect bone remodeling were excluded. Since the levels of *Cxcl12* and *CCcl5* mRNA in human bone samples had not been assessed when we started the study, we were unable to use a power analysis to determine the number of samples that would be required. We estimated that we would require a minimum of 15 samples from children and from adults to detect statistically significant differences in levels between them.

### ChIP assay

Transcription factor binding sites within −1 kb before the start codon of the murine *Cxcl12* gene were searched using TFSEARCH software, and 1 κB binding site was predicted in this region on the *Cxcl12* gene promotor. The binding of RelA and RelB to this κB binding site was tested by ChIP assays following the procedure we published.^14^ Briefly, the sheared chromatin from TRAF3^f/f^ (WT) and Prx1^Cre^;TRAF3^f/f^ (P-cKO) BdMPCs that had been fixed with 1% formaldehyde was immunoprecipitated with anti-RelA or -RelB Abs, or rabbit IgG as a negative control. The precipitated DNA was used as a template for PCR using primers specifically designed to amplify a segment of 150–250 bp covering the putative κB binding site. The sequences of the primers are: *Cxcl12* site 1, forward 5′-GACACTGAGGGCGCCAAGAA-3′ and reverse 5′-GCGCTTTAGAGGCGAAAACC -3. In addition, two pairs of unrelated primers were designed as control in the DNA region that is away from the κB binding site on the *Cxcl12* gene promotor. The primer sequences were as follows: pair 1, forward 5′-TGGTCGAAGCATGGCCAAC-3′ and reverse 5′-CATTGGTGTTCAGCCCTGC-3; and pair 2, forward 5′-GTGTCCAGCTGAGATGCACT-3′ and reverse 5′-GTGCCCACCAAGGTACAGAG-3. The procedure for quantitative real-time PCR described above was next performed to analyze the relative levels of RelA and RelB binding to the κB binding site.

### Cell migration assay

Migration was determined by using a transwell migration assay with a pore size of 0.5 μm on the membrane (Corning, Cata #: 3421), according to the manufacturer’s instructions. 3rd passage WT and P-cKO MPCs were seeded in 24-well plates at a concentration of 5 × 10^4^ per well and maintained for 2 d without medium change. Meanwhile, RANKL^+^B220^hi^IgM^+^ cells sorted from young and aged BM were treated in 1% heat-inactivated fetal bovine serum (FBS) with 50 μmol/L plerixafor for 2 h. 3 × 10^4^ of these pre-treated cells were resuspended in 100 μL RPMI 1640 medium containing 1% FBS plus 50 μmol/L plerixafor and added into each transwell insert. The inserts then were placed in the lower chambers, which were coated with MPCs. After incubation for 6 h, the inside of each inset was gently rubbed with cotton swabs to remove cells that failed to migrate into the membrane, following the manufacturer’s instructions. Following crystal violet staining, cells maintained on the bottom side of the membranes were counted using a light microscope.

### Radiography

Tibiae and spines were dissected from TGFβRII^fl/fl^ and TGFβRII^fl/fl^Prx1^Cre^ mice and soft tissues were removed. After fixation in 10% neutral buffered formalin for 2 d, these bones were imaged using a Faxitron Cabinet X-ray System (Faxitron Bioptics LLC., Tucson) with a setting of 30 KV, 5 s.

### Liver and renal toxicity assays

Fresh blood samples were collected and immediately placed into tubes containing a coagulation-promoting agent. The tubes were then incubated on ice for 1 h to facilitate clotting. Following incubation, the samples were centrifuged at 3 000 r/min for 10 min at 4 °C. Samples with significant hemolysis were noted and excluded from further analysis. Subsequently, 200 μL of the supernatant serum was carefully transferred to a new vessel for biochemical analysis. Serum levels of key biochemical indicators, including alanine aminotransferase, aspartate aminotransferase, blood urea nitrogen and creatinine, were measured using a BK-600 Discrete Fully Automated Biochemistry Analyzer (Boke Biotechnology Co., Ltd., Shandong, China).

### Statistics

All results are given as the mean ± S.D. Variance was similar between groups for most parameters assessed. Comparisons between two groups were analyzed using Student’s two-tailed unpaired *t* test and those among 3 or more groups using ANOVA followed by Tukey’s post-hoc multiple comparisons. *P* values < 0.05 were considered statistically significant. Each experiment was repeated at least twice with similar results. The sample size for in vivo experiments is based on an un-paired *t*-test power analysis carried out by our statistician using SigmaStat Statistical Software: 5–8 mice were needed in each group where bone parameters were being assessed to detect significant differences from controls with an alpha error of 5%. The power is 0.98, i.e., there is 98% chance of detecting a specific effect with 95% confidence when alpha = 0.05. No data were excluded from the analyses.

## Supplementary information

Supplementary Figures

## Data Availability

All relevant data are available from the authors upon reasonable request.
