# Supplementary material for: A RANKL+/CXCR4+ B cell population accumulates in bone marrow and causes age-related osteoporosis in mice
Source: Bone Res. 2026 May 18;14:53. doi: 10.1038/s41413-026-00525-5 (PMC13183972; doi:10.1038/s41413-026-00525-5)
Supplement: Supplementary file 1 — Supplementary Figures [file 41413_2026_525_MOESM1_ESM.pdf]

Supplementary Figure 1

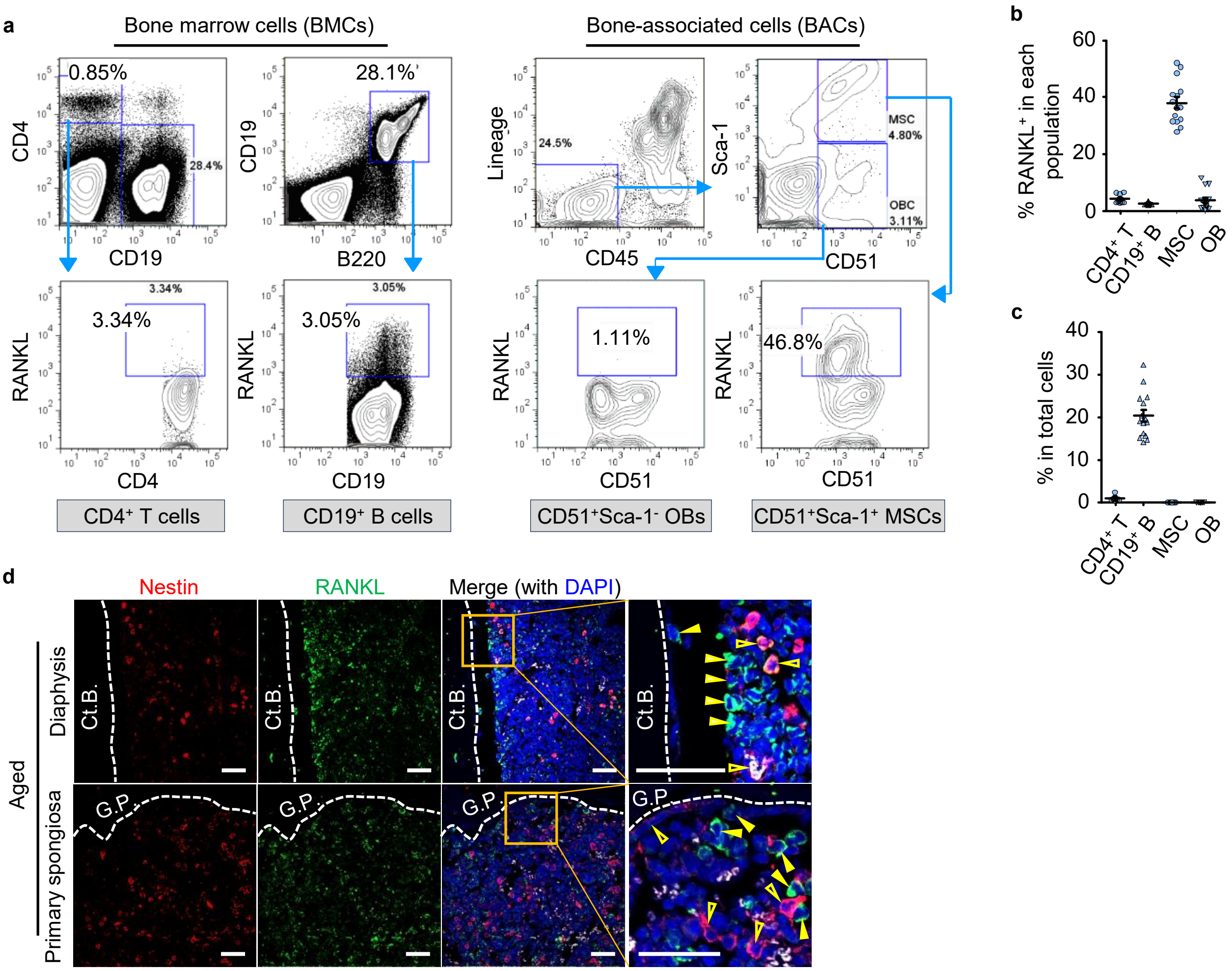

Supplementary Figure 1. **RANKL expression by BM and bone-associated cells**. (**a**) Representative graphs showing flow gates of various cell types in murine tibial and femoral bone and their expression of RANKL. (**b**) % RANKL$^+$ cells in cell types, including CD4$^+$ T and CD19$^+$ B cells in bone marrow cells (BMCs), and osteoblastic cells (OB) in bone-associated cells (BACs), and mesenchymal stromal cells (MSC) in total cells (BMCs plus BACs digested from bone). Mean ± SD (n=15 mice). (**c**) % each cell type in (**b**). Mean ± SD (n=15 mice). (**d**) Representative images showing Nestin- and RANKL-expressing cells in diaphysis (where cells have retracted from the bone surface during cutting) and primary spongiosa of tibiae from aged mice. Nestin$^+$ cells (red) and RANLK$^+$ cells (green) are indicated by yellow hollow and solid triangles, respectively. Bar=25 μm. G.P., growth plate; Ct.B., cortical bone.

Supplementary Figure 2

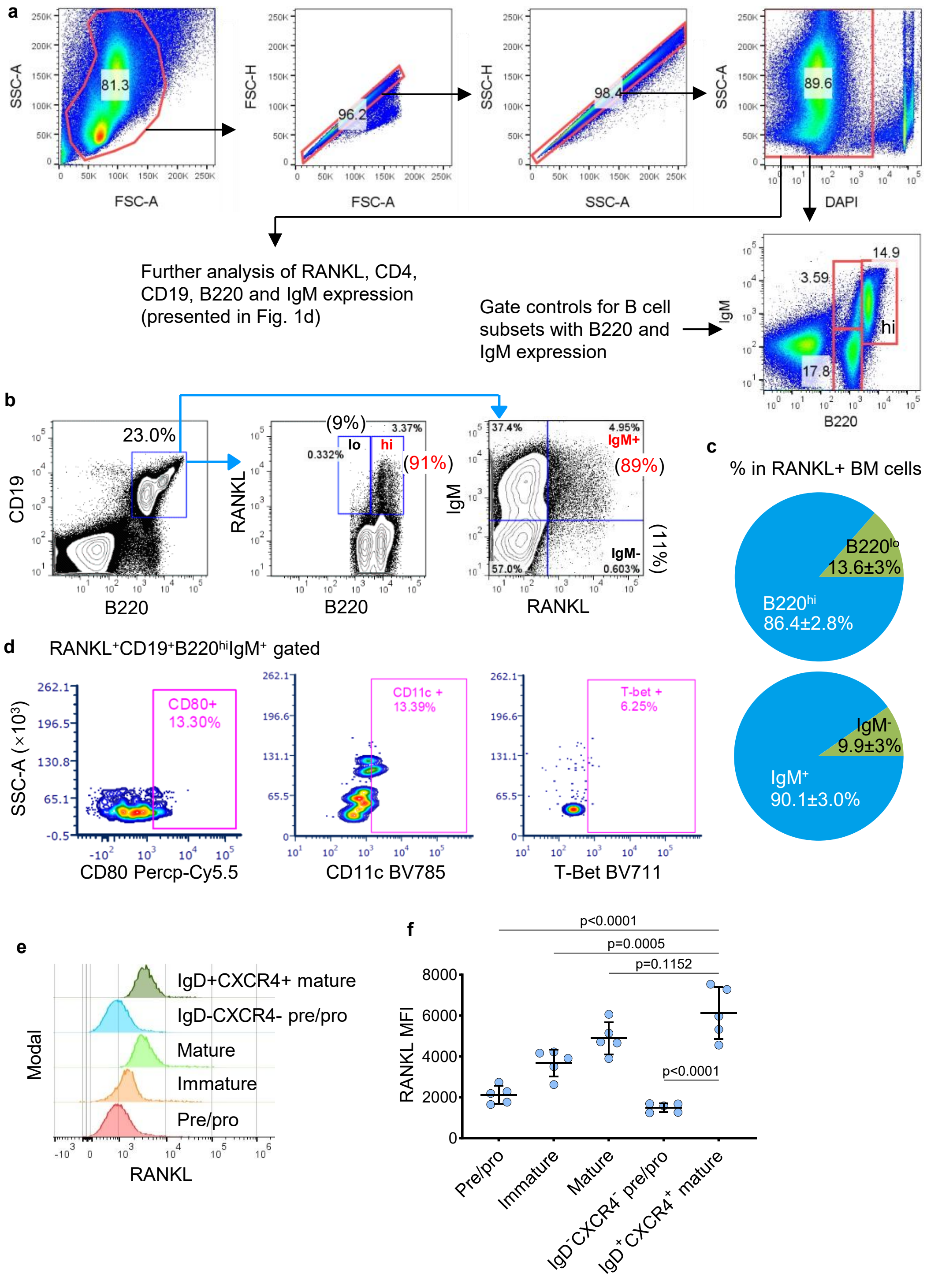

Supplementary Figure 2. **RANKL expression by B220^{hi}IgM^{+} B cells**. (**a**) Representative images showing the flow gates for live BM cell singlets for further analysis of RANKL, CD4, CD19, B220 and IgM expression. (**b**) Flow graphs showing the distribution of RANKL-expressing cells in B220^{hi} and B220^{lo}, as well as IgM^{+} and IgM^{-} B cells. (**c**) Pie charts of % RANKL^{+} cells in these B cell subpopulations. Mean ± SD (n=15 mice). (**d**) Representative images showing the expression of CD80, CD11c and T-Bet by RANKL^{+}CD19^{+}B220^{hi}IgM^{+} cells. (**e**) Histogram graphs of RANKL expression by various B cell subsets, and (**f**) mean fluorescent intensity (MFI) of RANKL by different B cell subsets in BM. Mean ± SD (n=5 mice). Analysis: One-way ANOVA with Tukey's post hoc test in (**f**).

Supplementary Figure 3

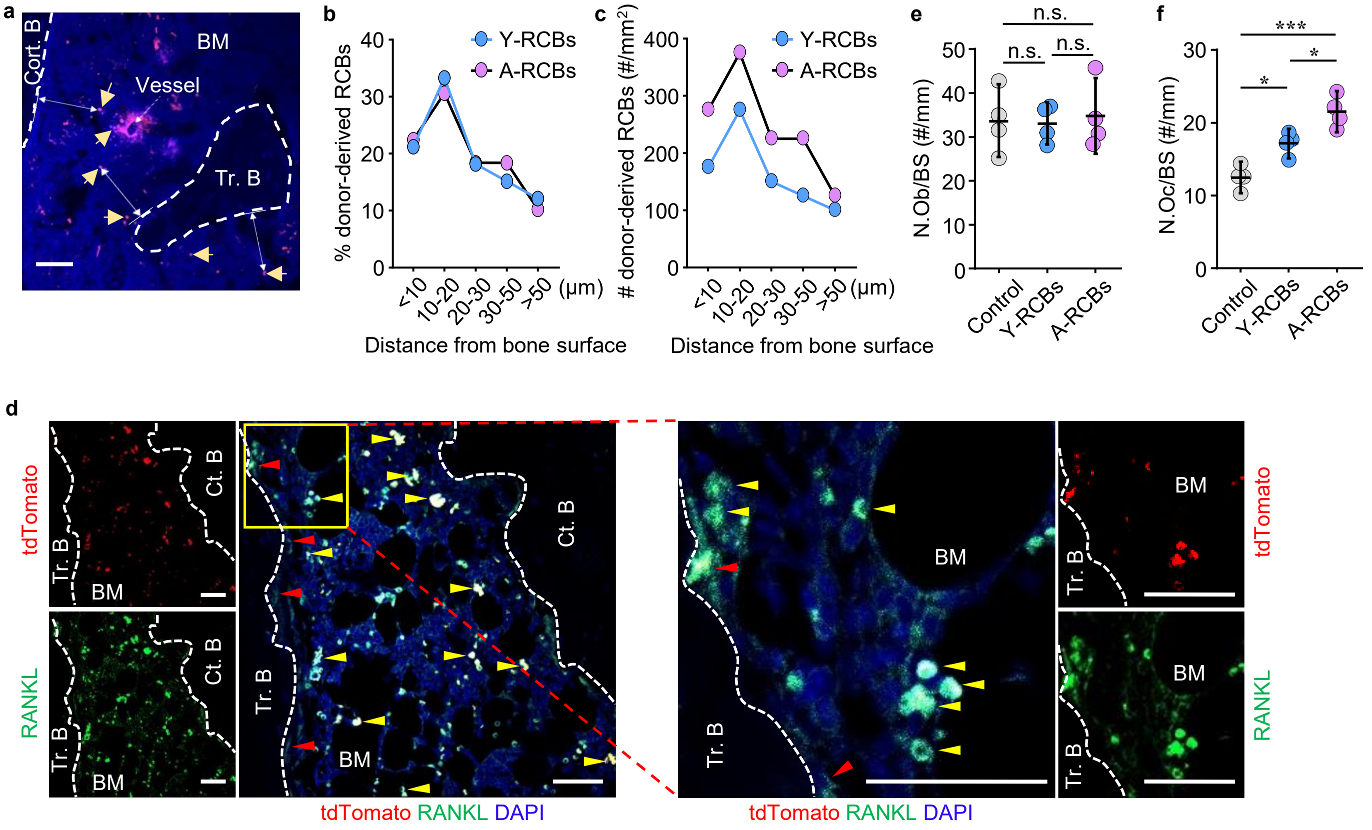

Supplementary Figure 3. **Donor-derived RCBs in bone of NSG recipient mice associated with increased osteoclast numbers**. (**a**) Representative immunofluorescence image of a bone section from a NSG mouse injected with donor-derived RCBs (tdTomato^{+} yellow arrows). Cort. B: cortical bone. Tr. B: trabecular bone. BM: bone marrow. Bar=25 µm. (**b-c**) % and numbers of donor-derived RCBs, based on the distance of RCBs (tdTomato^{+}) to the nearest bone surface. (**d**) Immunofluorescence images of bone cryosections showing RANKL expression by osteoblastic cells (green) on bone surfaces (indicated by red triangles), aged mouse donor-derived RCBs (tdTomato red; indicated by yellow triangles) and other BM cells. Ct. B: cortical bone. Tr. B: trabecular bone. BM: bone marrow. Bar=50 µm. (**e-f**) Numbers of osteoblasts on bone surfaces in H&E-stained sections and of osteoclasts on bone surfaces in TRAP-stained paraffin sections from NSG recipient mice. Mean ± SD (n=4 mice/group). Analysis: One-way ANOVA with Tukey's post hoc test in (**e**) and (**f**). *p<0.05; ***p<0.001.

Supplementary Figure 4

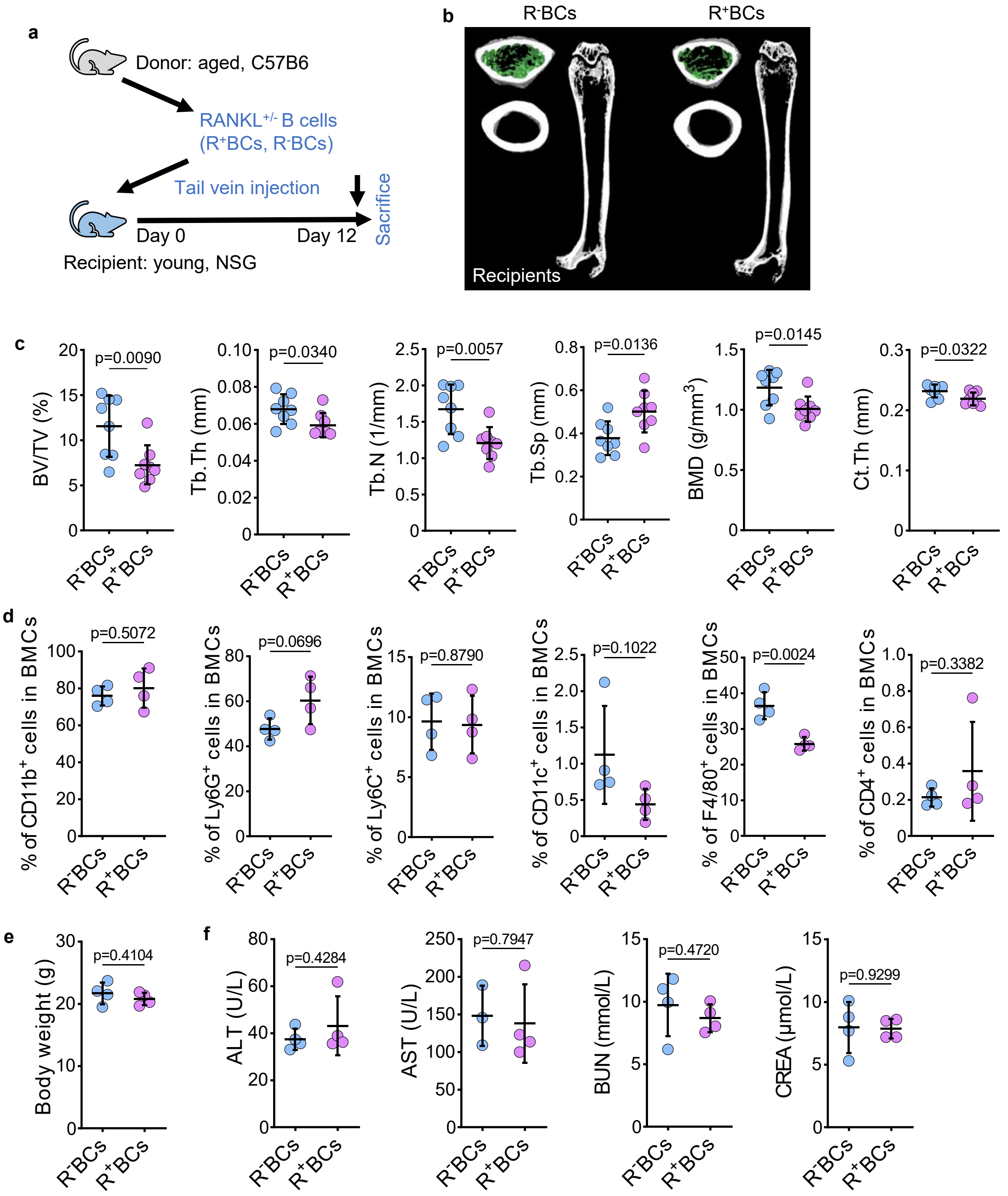

Supplementary Figure 4. **BM RANKL$^+$ B cells from aged mice cause bone loss and a decrease in macrophages in BM of young NSG mice**. (**a**) Cartoon showing the procedure for cell transplantation: sorted RANKL$^-$B220^{hi}IgM$^+$ B cells (R$^-$BCs) and RANKL$^+$B220^{hi}IgM$^+$ B cells (R$^+$BCs) from BM of aged C57 male mice were injected into the tail veins of 3-mon-old male NSG mice and the recipients were sacrificed 12 d post transfer. (**b**) Representative µCT 3D images of femora from 3-m-old NSG recipient mice injected with R$^-$BCs or R$^+$BCs from aged mice, and (**c**) analysis of microstructure parameters, including trabecular bone volume (BV/TV), thickness (Tb.Th), number (Tb.N), separation (Tb.Sp), bone mineral density (BMD) and cortical bone thickness (Ct.Th) values. Mean ± SD (n=8 mice/group). (**d**) The percentages of various types of BM cells in NSG recipient mice tested by flow. Mean ± SD (n=4 mice/group). (**e-f**) Mouse body weight and liver and renal functional parameters, including serum alanine aminotransferase (ALT), aspartate aminotransferase (AST), blood urea nitrogen (BUN) and creatinine (CREA) levels. Mean ± SD (n=4 mice/group). Analysis: Student's two-sided unpaired *t* test.

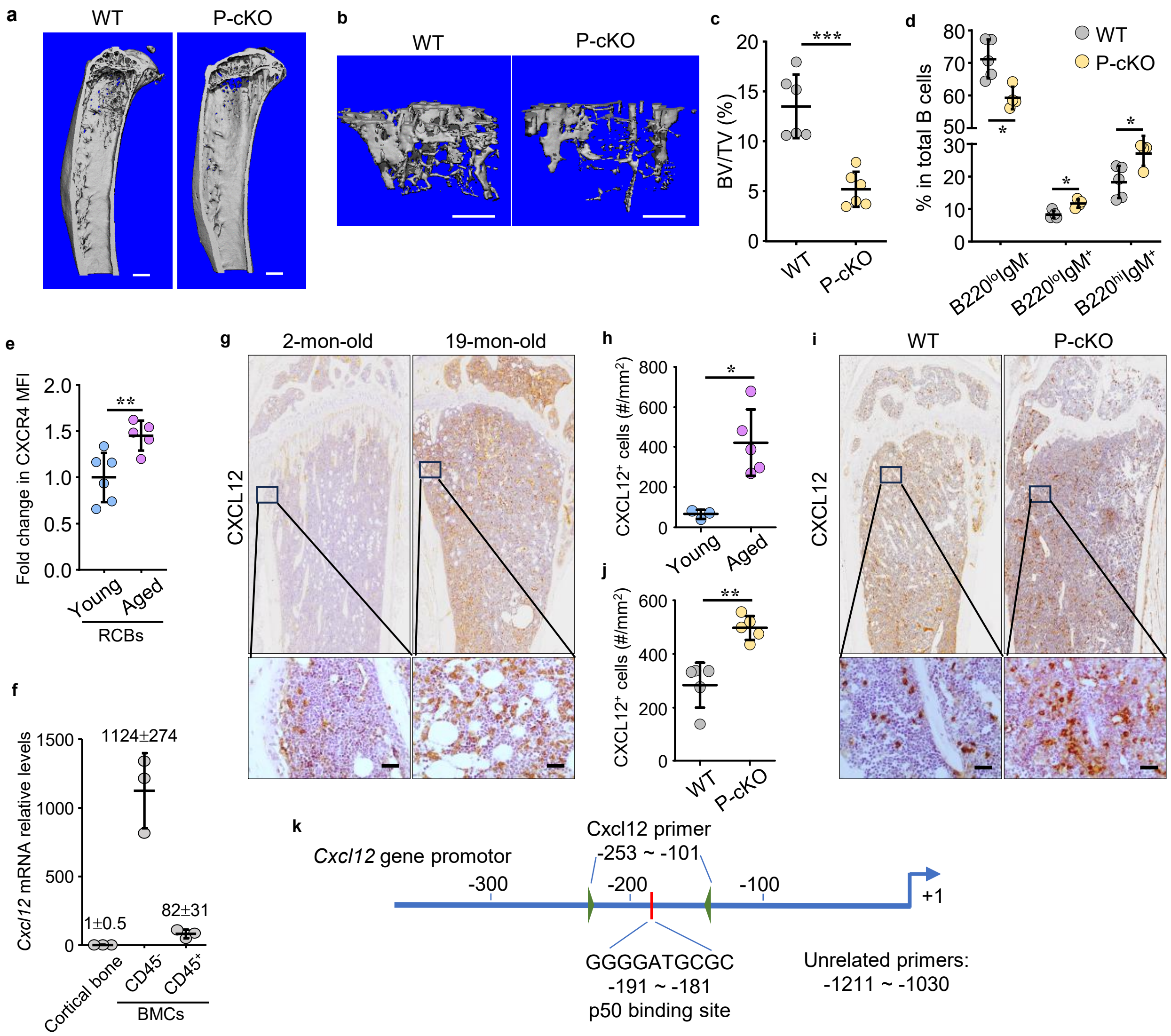

Supplementary Figure 5. **Adult P-cKO mice have increased RCBs and CXCL12-expressing cells in BM**. (**a, b**) Low and high power µCT 3D images of tibiae from 12-mon-old male TRAF3$^{fl/fl}$ (WT) and Prx1^{Cre}TRAF3$^{fl/fl}$ (P-cKO) mice. Bar=500 µm. (**c**) Tibial trabecular bone volume (BV/TV) levels in 12-mon-old male WT and P-cKO mice. Mean ± SD (n=6 mice/group). (**d**) % of B cell subsets in total number of B cells in BM from 12-mon-old male WT and P-cKO mice. Mean ± SD (n=5 WT and 4 P-cKO mice). (**e**) Fold changes in mean fluorescence intensity (MFI) of CXCR4 expression on RCBs in BM from young and aged mice. Mean ± SD (n=6 young and 5 aged mice). (**f**) *Cxcl12* mRNA levels in cortical bone (with BM flushed out) and magnetically-sorted BM CD45$^-$ and CD45$^+$ cells from 3-mon-old C57Bl/6J mice. Mean ± SD (n=3 mice). (**g**) Representative images of CXCL12 immunohistochemistry in tibial paraffin sections from 2- and 19-mon-old C57Bl/6J mice and (**h**) numbers of CXCL12$^+$ cells in their tibial metaphyses. Bar=25 µm. Mean ± SD (n=3 young and 5 old mice). (**i-j**) CXCL12 immunohistochemistry in tibial paraffin sections from 15-mon-old WT and P-cKO mice and (**j**) numbers of CXCL12$^+$ cells in their tibial metaphyses. Bar=25 µm. Mean ± SD (n=5 mice/group). (**k**) Scheme for mouse *Cxcl12* gene promoter analysis showing a putative κB binding site and primer design to test the binding site. Analysis: Student's two-sided unpaired *t* test. * $p<0.05$; ** $p<0.01$; *** $p<0.001$.

Supplementary Figure 6

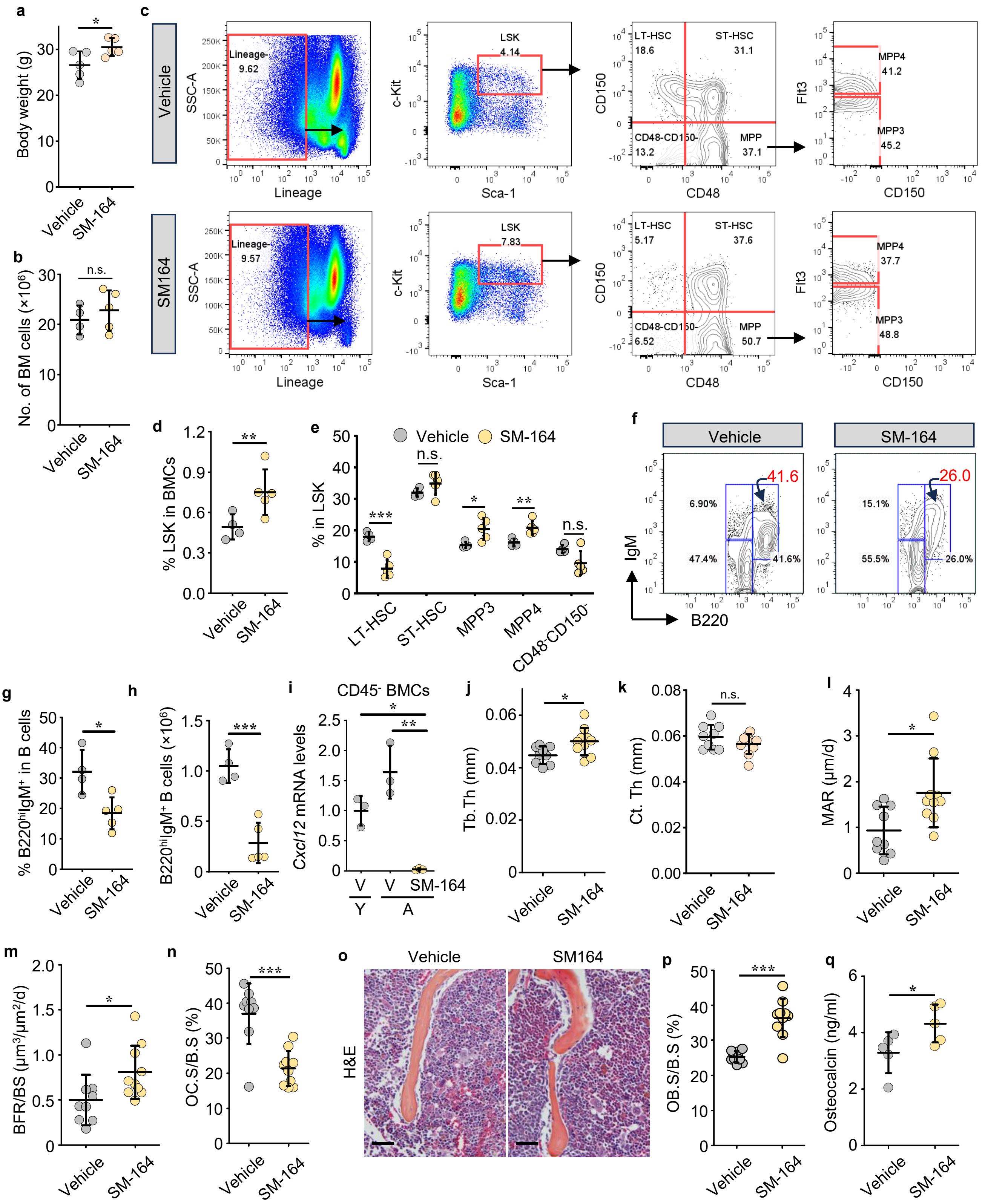

Supplementary Figure 6. **SM-164-treated aged mice have fewer B220^{hi}IgM^{+} recirculating B cells, lower Cxcl12 transcription, and increased osteoblastic bone formation.** (**a-b**) Body weights of 22-mon-old (aged) male C57Bl/6J mice treated with vehicle or SM-164 (3 mg/Kg/day) once/d for one month. Mean ± SD (n=5 vehicle- (gray circles) and 5 SM-164-treated (yellow circles) mice. (**b**) Numbers of total BM cells in one femur plus two humeri from each mouse. Mean ± SD (n=4 vehicle- and 5 SM-164-treated mice). (**c**) FACS analysis of hematopoietic stem and progenitor cells in BM of mice. (**d**) % of Lineage^{-}Sca1^{+}cKit^{+} (LSK) cells in total BM cells in mice. Mean ± SD (n=4 vehicle- and 5 SM-164-treated mice). (**e**) % of long- and short-term hematopoietic stem cells (LT-/ST-HSC) and multipotent progenitors (MPP) in LSK cells from BM of mice. Mean ± SD (n=4 vehicle- and 5 SM-164-treated mice). (**f**) FACS gates of B cell subpopulations, including pro/pre (B220^{lo}IgM^{-}), immature (B220^{lo}IgM^{+}) and recirculating (B220^{hi}IgM^{+}) B cells in BM. (**g-h**) % and numbers of B220^{hi}IgM^{+} recirculating B cells. Mean ± SD (n=4 vehicle- and 5 SM-164-treated mice). (**i**) *Cxcl12* mRNA levels in magnetically-sorted BM CD45^{-} cells from young (Y; 3-mon-old) and aged (A; 22-mon-old) C57Bl/6J mice treated with vehicle or SM-164. Mean ± SD (n=3 mice/group). (**j-k**) Trabecular and cortical bone thickness in μCT scans, and (**l-m**) mineral apposition rate (MAR) and bone formation rate (BFR) of vertebrae from 22-mon-old (aged) male mice treated with vehicle and SM-164. Mean ± SD (n=9 vehicle- and 10 SM-164-treated aged mice). (**n**) OC surfaces in TRAP-stained paraffin vertebral sections of mice treated with vehicle and SM-164. (**o-p**) Representative H&E-stained paraffin sections of L2 vertebrae (**o**) and osteoblast surfaces (**p**) in 9 vehicle- and 10 SM-164-treated aged mice. Bar=50 μm. (**q**) Serum osteocalcin tested by ELISA. Mean ± SD (n=5 mice/group). Analysis: One-way ANOVA with Tukey's post hoc test in (**i**); Student's two-sided unpaired *t* test in others. *p<0.05; **p<0.01; ***p<0.001.

Supplementary Figure 7

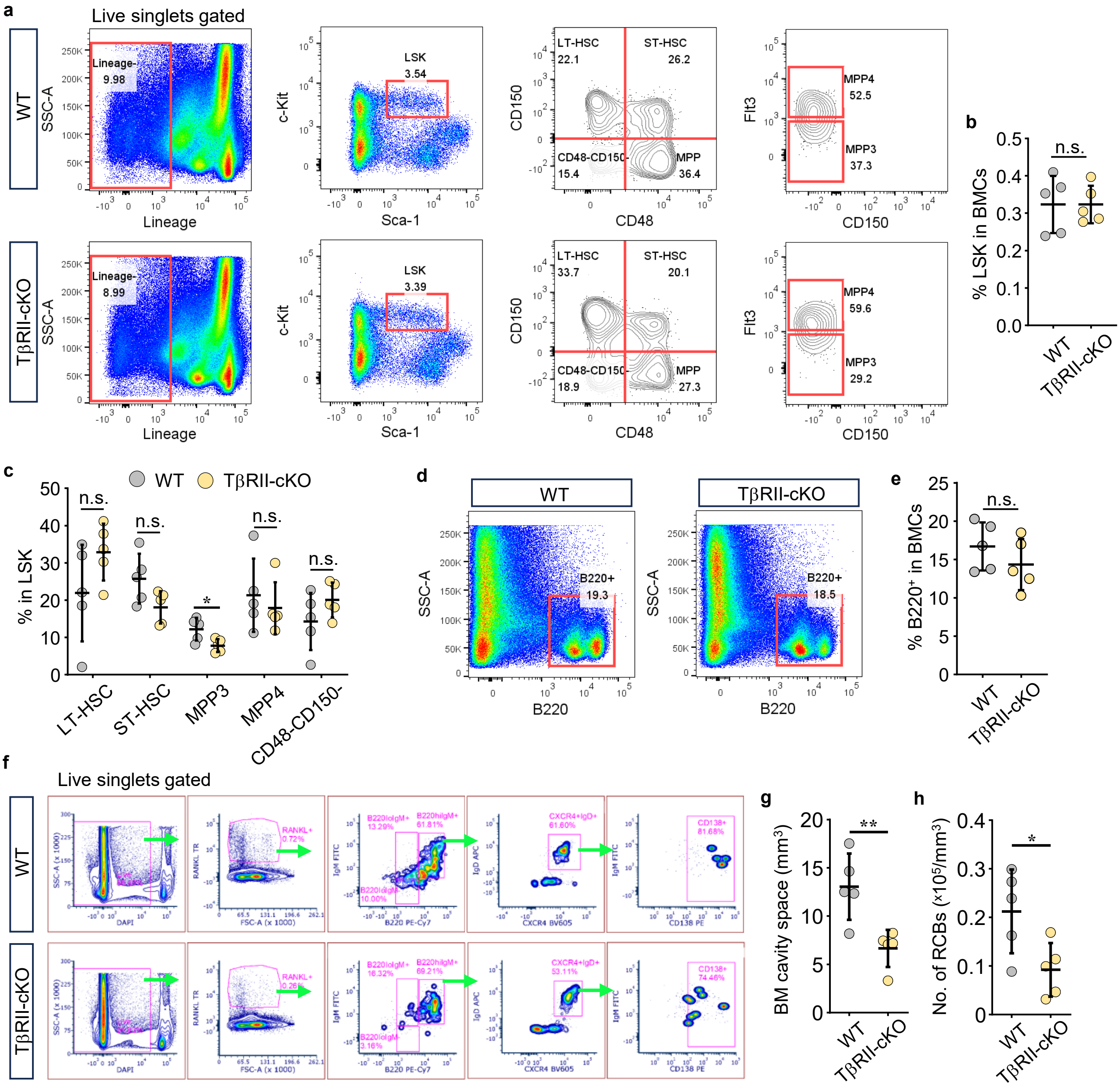

Supplementary Figure 7. **Mice with TGFβRII specific deletion in mesenchymal lineage cells have normal B cell generation but decreased RCB numbers in BM.** (**a**) FACS analysis of hematopoietic stem and progenitor cells in BM of 15-mon-old TGFβRII$^{fl/fl}$ (WT) and Prx1^{Cre}TGFβRII$^{fl/fl}$ (TβRII-cKO) mice. (**b**) % of Lineage^{-}Sca1^{+}cKit^{+} (LSK) cells in total BM cells. Mean ± SD (n=5 mice/group). (**c**) % of long-/short-term hematopoietic stem cells (LT-/ST-HSC) and multipotent progenitors (MPP) in LSK cells from BM of WT and TβRII-cKO mice. Mean ± SD (n=5 mice/group). (**d**) FACS analysis of B220$^{lo/hi}$ cells in BM of WT and TβRII-cKO mice, and (**e**) % of B220^{+} B cells in BM cells. Mean ± SD (n=5 mice/group). (**f**) FACS gates of RANKL^{+}B220^{hi}IgM^{+} B cell subset and characterization of their expression of IgD, CD138 and CXCR4. (**g**) BM cavity area in femora, based on the parameters in reconstructed µCT scans. Mean ± SD (n=5 mice/group). (**h**) Numbers of RCBs normalized to the BM cavity space of each mouse. Mean ± SD (n=5 mice/group). Analyses: Student's two-sided unpaired *t* test. *p<0.05; **p<0.01.

Supplementary Figure 8

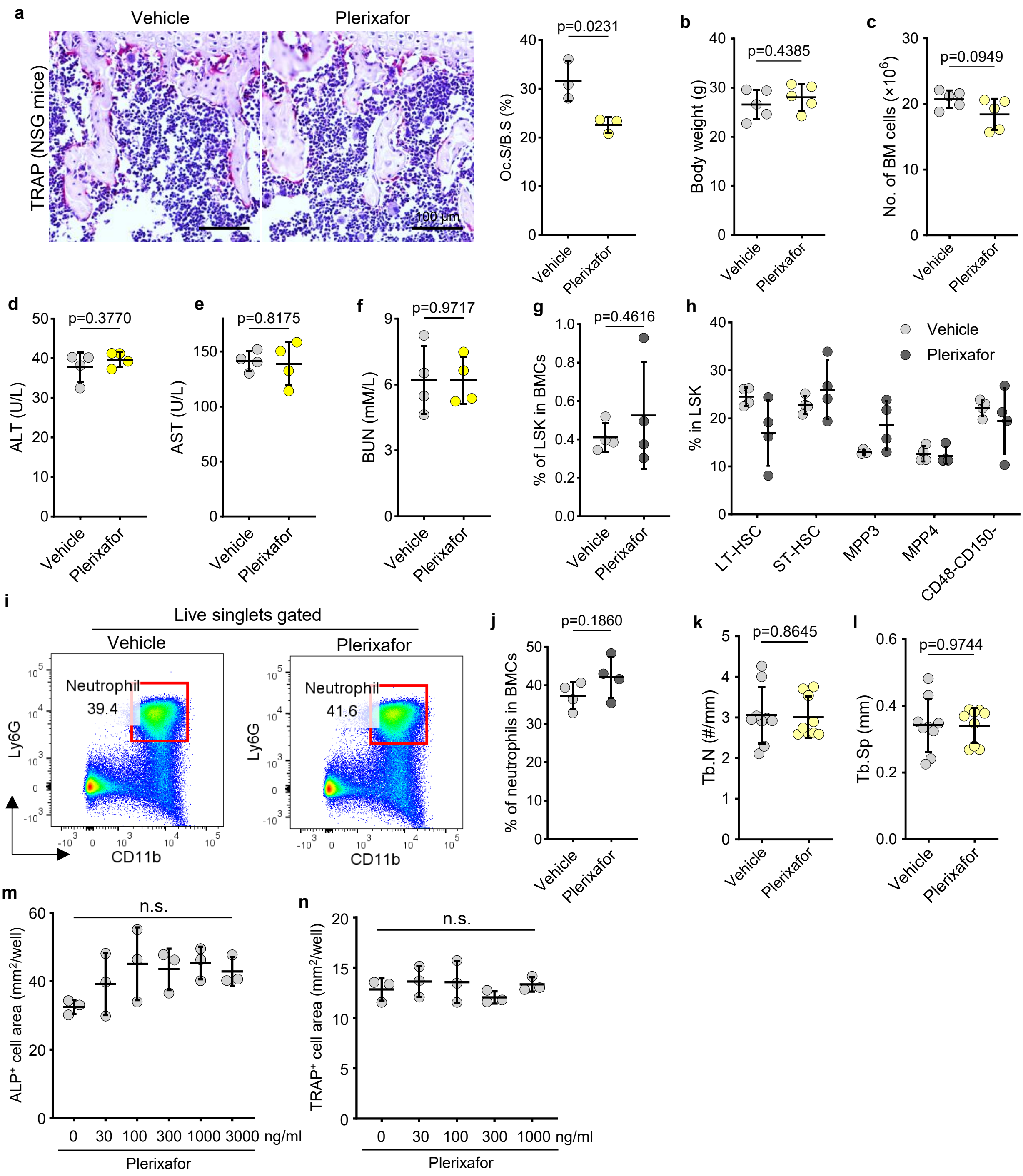

Supplementary Figure 8. **Changes in mice and cells following plerixafor administration**. (**a**) Representative images of TRAP-stained tibial paraffin sections from NSG mice injected with RCBs from aged mice and treated with vehicle or plerixafor, and osteoclast surfaces. Bar=100 μm. (**b-c**) Body weight and numbers of total BM cells from a tibia and pelvis of aged mice treated with vehicle or plerixafor. Mean ± SD (n=5 mice/group). (**d-f**) Concentration of serum alanine aminotransferase (ALT), aspartate aminotransferase (AST) and blood urea nitrogen (BUN) to assess liver and renal toxicity in mice in (**b**). Mean ± SD (n=4 mice/group). (**g**) % of Lineage$^-$ Sca1$^+$cKit$^+$ (LSK) cells in total BM cells in mice in (**b**). Mean ± SD (n=4 mice/group). (**h**) % of long- and short-term hematopoietic stem cells (LT-/ST-HSC) and multipotent progenitors (MPP) in LSK cells from BM of mice in (**b**). Mean ± SD (n=4 mice/group). (**i-j**) Representative images showing flow gates for CD11b$^+$Ly6G$^+$ neutrophils and % of neutrophils in BM of vehicle- and plerixafor-treated aged mice. (**k-l**) Vertebral μCT microstructure parameters, trabecular number (Tb.N) and separation (Tb.Sp). Mean ± SD (n=9 mice/group). (**m-n**) Osteoblast and osteoclast progenitor cells treated with increasing doses of plerixafor during differentiation and alkaline phosphatase (ALP)- and TRAP-positive areas, respectively, were counted. Analysis: Student's two-sided unpaired *t* test in a-l; One-way ANOVA with Tukey's post hoc test in (**m**) and (**n**).

Supplementary Figure 9

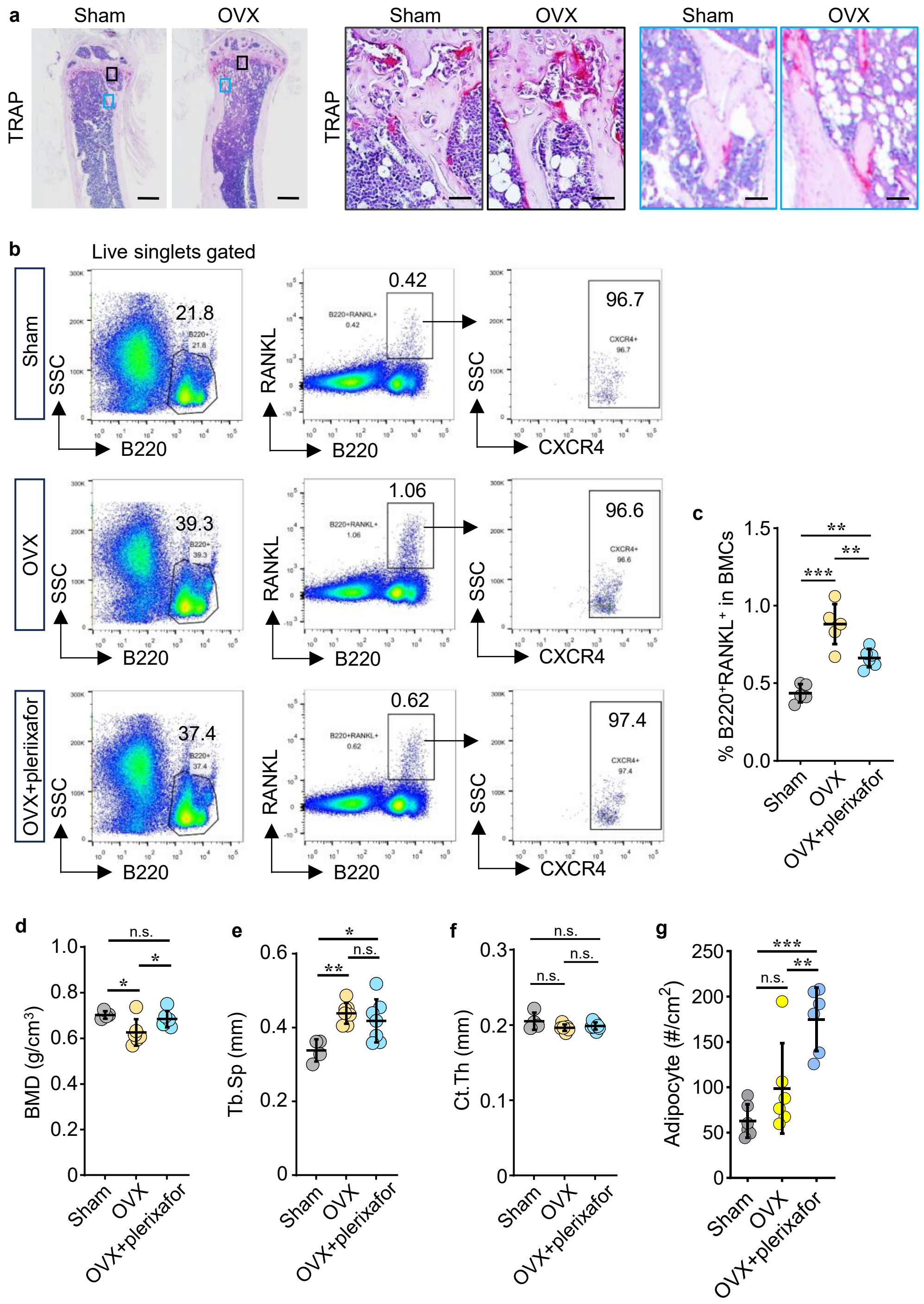

Supplementary Figure 9. **Blockade of CXCR4 prevents accumulation of RCBs in BM and bone loss in OVX mice.** (**a**) Representative images of TRAP-stained paraffin sections of tibiae from sham and OVX mice and the boxed areas at a higher magnification in the right panel. Bar=500 µm in the left panel and 25 µm in the right panel. (**b**) Representative images showing the gating strategies for B cells and RCBs in BM cells from sham, vehicle-treated OVX and plerixafor-treated mice. (**c**) % of RANKL^{+}B220^{+} cells in total BM cells. Mean ± SD (n=5, 5 and 6 C57Bl/6J mice for sham, OVX and OVX+plerixafor groups, respectively). (**d-f**) Analysis of bone mineral density (BMD, **d**), trabecular separation (Tb.Sp, **e**) and cortical bone thickness (Ct.Th, **f**) based on µCT 3D reconstruction of femora from sham and OVX mice treated with vehicle or plerixafor. Mean ± SD (n=4, 7, 7 mice for sham, OVX and OVX+plerixafor groups, respectively). (**g**) The numbers of adipocytes counted in H&E-stained tibial paraffin sections. Analysis: One-way ANOVA with Tukey's post hoc test. *p<0.05; **p<0.01; ***p<0.001.
